# Four functional profiles for fibre and mucin metabolism in the human gut microbiome

**DOI:** 10.1186/s40168-023-01667-y

**Published:** 2023-10-20

**Authors:** Simon Labarthe, Sandra Plancade, Sebastien Raguideau, Florian Plaza Oñate, Emmanuelle Le Chatelier, Marion Leclerc, Beatrice Laroche

**Affiliations:** 1 Université Paris-Saclay, INRAE, MaIAGE, 78350 Jouy-en-Josas, France; 2 Univ. Bordeaux, INRAE, BIOGECO, 33610 Cestas, France; 3 Inria, INRAE, Pléiade, 33400 Talence, France; 4 UR875 MIAT, Université fédérale de Toulouse, INRAE, Castanet-Tolosan, France; 5 Earlham Institute, Organisms and Ecosystems, NR4 7UZ Norwich, UK; 6 Université Paris-Saclay, INRAE, MGP, 78350 Jouy-en-Josas, France; 7 Université Paris-Saclay, INRAE, Micalis, 78350 Jouy-en-Josas, France; 8 Pendulum Therapeutics, San Francisco, USA; 9 Inria, INRAE, Musca, 91120 Palaiseau, France

**Keywords:** Metagenomics, NMF, Functional profiling, Statistical learning

## Abstract

**Background:**

With the emergence of metagenomic data, multiple links between the gut microbiome and the host health have been shown. Deciphering these complex interactions require evolved analysis methods focusing on the microbial ecosystem functions. Despite the fact that host or diet-derived fibres are the most abundant nutrients available in the gut, the presence of distinct functional traits regarding fibre and mucin hydrolysis, fermentation and hydrogenotrophic processes has never been investigated.

**Results:**

After manually selecting 91 KEGG orthologies and 33 glycoside hydrolases further aggregated in 101 functional descriptors representative of fibre and mucin degradation pathways in the gut microbiome, we used nonnegative matrix factorization to mine metagenomic datasets. Four distinct metabolic profiles were further identified on a training set of 1153 samples, thoroughly validated on a large database of 2571 unseen samples from 5 external metagenomic cohorts and confirmed with metatranscriptomic data. Profiles 1 and 2 are the main contributors to the fibre-degradation-related metagenome: they present contrasted involvement in fibre degradation and sugar metabolism and are differentially linked to dysbiosis, metabolic disease and inflammation. Profile 1 takes over Profile 2 in healthy samples, and unbalance of these profiles characterize dysbiotic samples. Furthermore, high fibre diet favours a healthy balance between profiles 1 and profile 2. Profile 3 takes over profile 2 during Crohn’s disease, inducing functional reorientations towards unusual metabolism such as fucose and H2S degradation or propionate, acetone and butanediol production. Profile 4 gathers under-represented functions, like methanogenesis. Two taxonomic makes up of the profiles were investigated, using either the covariation of 203 prevalent genomes or metagenomic species, both providing consistent results in line with their functional characteristics. This taxonomic characterization showed that profiles 1 and 2 were respectively mainly composed of bacteria from the phyla *Bacteroidetes* and *Firmicutes* while profile 3 is representative of *Proteobacteria* and profile 4 of methanogens.

**Conclusions:**

Integrating anaerobic microbiology knowledge with statistical learning can narrow down the metagenomic analysis to investigate functional profiles. Applying this approach to fibre degradation in the gut ended with 4 distinct functional profiles that can be easily monitored as markers of diet, dysbiosis, inflammation and disease.

Video Abstract

**Supplementary Information:**

The online version contains supplementary material available at 10.1186/s40168-023-01667-y.

## Background

The generalization of metagenome sequencing 15 years ago has provided ample evidence of the complex interactions between the gut microbiota and its host health [1]. Since then, a large number of new links between the function and composition of the microbiota and the host health have been consistently discovered [2]. Significant efforts put into the recruitment of large cohorts to constitute reference datasets made it possible to explore the high inter-individual variability of the microbial communities in the gut [3–6].

Metabarcoding methods have been first popularized. Amplification of universal taxonomic marker gene before sequencing allows the construction of taxonomic entities (Operational Taxonomic Units, OTUs [7], or Amplicon Sequence Variants, ASVs [8]) informing on the phylogenetic composition of the microbial community [9] and on ecological biomarkers such as diversity indices [10]. Additional analysis can show co-occurrence networks [11, 12] or dynamical interactions in time-series [13, 14] both informing on ecological interactions. However, as the functional potential of the microbial populations remains unknown with metabarcoding techniques, the functional mechanisms that drive these interactions cannot be identified, even if tools leveraging reference databases of known genomes partially mitigate this issue [15].

With the development of metagenomic next-generation sequencing (mNGS) techniques [16], the entire functional information contained in the metagenomes became accessible. Shotgun sequencing together with bioinformatics methods identifying contigs between the sequenced fragments [17] and the constitution of massive catalogs of annotated genes [5] provide decisive tools for the study of the functional ecology in the gut microbiome. Multi-omics studies including metatranscriptomics or metabolomics give complementary information on the microbial functions actually activated in the gut [4]. Taxonomic and functional ecology can be addressed simultaneously with mNGS with the identification of entire microbial genomes in the metagenomes, such as metagenomic species (MGS [18]) or metagenome-assembled genomes (MAG [19]). Statistical analysis makes it possible to decipher universal MGS patterns in both metabarcoding and metagenomic cohorts, termed enterotypes, that are linked to different physiopathological status [20].

However, despite the massive amount of metagenomic data that were gathered by the microbial ecology community and the sophisticated agnostic data-driven analysis methods that were developed, the understanding of the mechanisms involved in the gut microbiota regulation and dynamics remains scarce. This observation calls for the development of new approaches operating a shift from descriptive ecology towards functional ecology [21] by leveraging existing knowledge in microbiology to explore the links between community structure and functions [22].

Dietary and host-derived fibres are the main primary substrate for the gut microbiota [23] so that anaerobic hydrolysis and consecutive downstream sugar degradation towards short-chain fatty acids (SCFAs) are the most common microbial functions in the colon, the distribution of which reflects the fibre intake [23]. The corresponding metabolic pathways are very well characterized [24], hence providing suitable candidate functions for pattern identification and differential analysis. Considering the well-defined framework of fibre anaerobic hydrolysis, we hypothesize that (H1) functional invariants can be deciphered, defining ‘universal’ functional profiles shared by all individuals, describing fibre degradation in the microbiota, (H2) functional and taxonomic interpretation of these profiles can be obtained and (H3) these profiles characterize the metagenomic samples and are related to dysbiosis or disease.

In this study, we build on a method proposed in [25], which informs a data-driven dimension reduction technique termed nonnegative matrix factorization (NMF) with the well-established knowledge of fibre degradation pathways in the gut to analyse fibre-degradation-related metagenomic count matrix. The method is trained on a database of 1152 samples and validated on 5 external databases gathering 2571 unseen samples, allowing to identify four functional Profiles the mixture of which reconstruct the metagenomes, the expression of which is confirmed with metatranscriptomics. Extensive functional and taxonomic characterization of the profiles is performed and systematic differential analysis is conducted to identify possible links between the profiles and the sample physiopathological status. The microbiota simplification provided by the method allows in-depth biological interpretations of the differential analysis.

## Methods

We first introduce the different datasets that are considered in this study. We then describe the rationale of the function selection and the pooling of the corresponding genes related to dietary and host-derived fibre degradation pathways, and the subsequent bioinformatics, from the samples to the frequencies matrix. We finally detail the NMF decomposition of the frequencies matrices to identify functional profiles in the metagenomes. Finally, we present the differential analysis method, based on Profiles weights in samples.

### Training and external validation datasets

A training set was assembled with $n_s=1126$ samples covering a balanced mix of health status, including healthy samples, inflammatory diseases (Crohn’s disease (CD), ulcerative colitis (UC)) and metabolic diseases (obese, type 2 diabetes) taken from 7 cohorts (accession ID PRJEB1220 [19], PRJEB4336 [26], PRJEB5224[5, 27], PRJNA48479 [28], PRJNA422434 [29], PRJEB6337 [30], PRJNA375935 [31]) and 5 countries (USA, China, Spain, Denmark, France) to avoid potential study or country effects. External validation datasets were taken from studies selected for their focus on a specific effect. We selected two cohorts dedicated to IBD―hmp2 (PRJNA398089 [4], $n_s=1266$ samples) and CD (PRJEB15371 [32], $n_s=119$ samples)―one cohort to obesity―metacardis (accession ID PRJEB37249 [33], $n_s=883$ samples)―one cohort to Mediterranean diet (accession ID PRJEB33500 [34], $n_s=244$) and one to Parkinson disease (accession ID PRJEB17784 [35], $n_s=59$ samples) since this disease is associated to a longer transit time and microbial modifications. Note that 3 and 5 samples, respectively, have been removed from cohorts PRJEB15371 and PRJEB37249 after quality checks. All together, these datasets make it possible to consider a large variety of co-variables, including dysbiosis index (DI, see the “Statistical treatment” section), body mass index (BMI) used to define obesity, statin treatment against hypercholesterolemia, the four enterotypes *Bacteroides* 1 (Bact1), *Ruminococcaceae* (Rum), *Prevotella* (Prev) and *Bacteroides* 2 (Bact2) [20, 33] and Bristol score [36] used to determine stool appearance. Dataset overview can be found in Table 1. Dataset homogeneity has been assessed by computing intra and inter-variability of pairwise Bray-Curtis distance (pBCd, see the “Statistical treatment” section and Fig. 2). The complete list of samples and their corresponding metadata can be found in Additional file 11 — dataset count matrices, profile decomposition and metadata.

**Table 1:** Dataset overview. We indicate for each dataset the number of samples $n_s$, individuals $n_i$, and if the dataset is used for DI, BMI, CD, statin, enterotypes, bristol score, diet or Parkinson studies

	train	hmp2	CD	metacardis	med.diet	Parkin.
$n_{s}$	1126	1266	119	883	244	59
$n_{i}$	1126	106	119	883	82	59
DI	x	x	x	x	x	x
BMI	x	x	x	x		
CD	x	x	x			
statin				x		
enter.				x		
Bristol				x		
diet					x	
Parkin.						x

### A functional view of fibre degradation in metagenomes

**Fig. 1:**
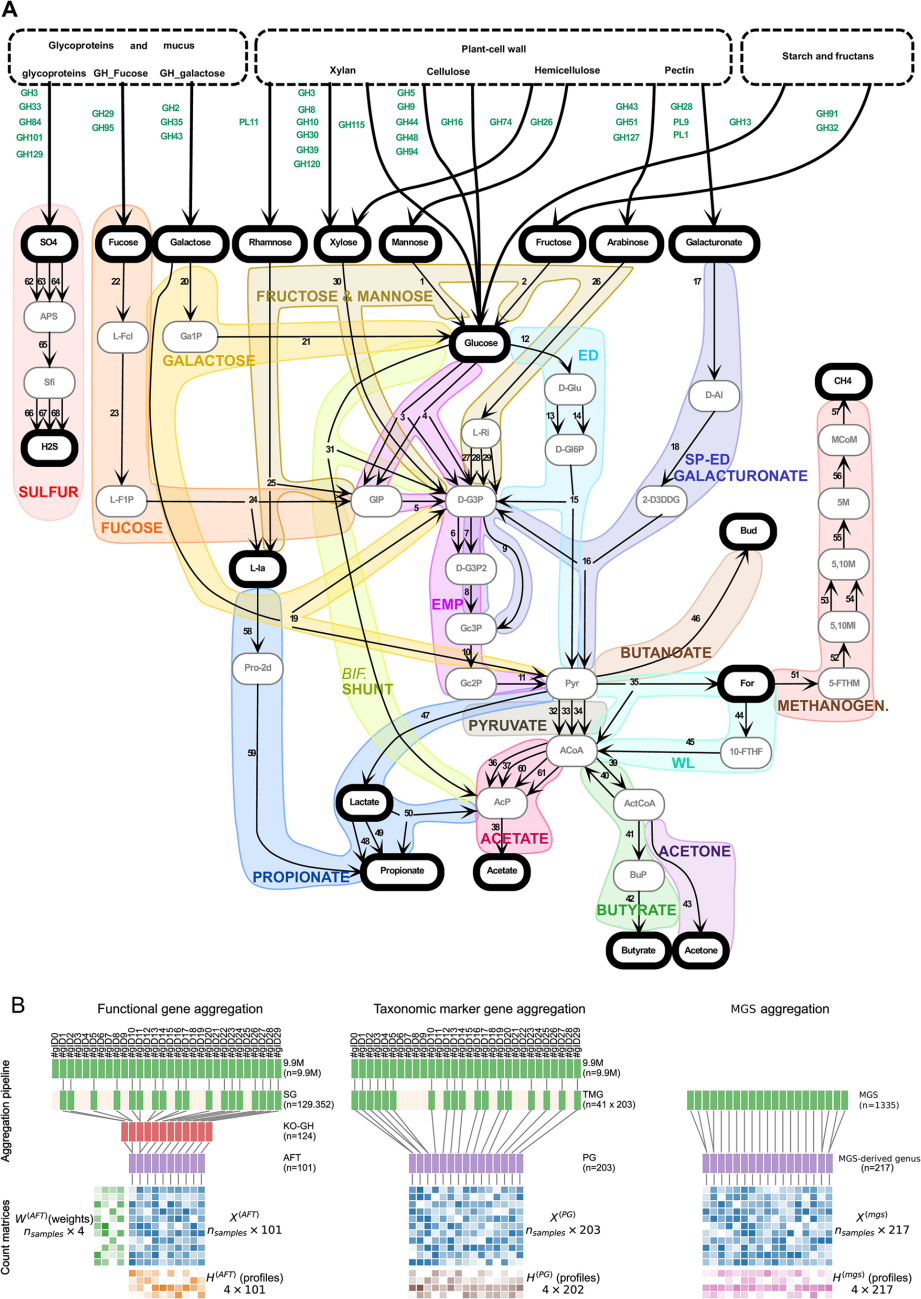
Modelling overview. **a** Schematic metabolic network of fibre degradation in the gut. The metabolic network used to model fibre degradation in the gut is represented from complex dietary and host-derived fibres to terminal metabolites. Dashed boxes in the upper part represent fibre pools that are linked to fibre-derived sugars by GH and PL. Intra- and extra-cellular metabolites are respectively represented by gray and black boxes. Metabolic pathways linking metabolites are numbered from 1 to 68 (see Table 2): representative KOs are selected for each pathway, checking for specificity (KO are not involved in other metabolic reactions) and essentialness (essential reactions for the completion of the pathway). Functional blocks are represented by colored shapes. GH_Fucose and GH_galactose, complex carbohydrate involving respectively fucose and galactose; ED, Entner-Doudoroff; SP-ED, semi-phosphorylative Entner-Doudoroff; EMP, Embden-Meyerhoff-Parnas; Bif. shunt, *Bifidobacterium* shunt; WL, Wood-Ljundhal. Complete list of reactions and abbreviations can be found in the Additional file 11—Dataset count matrices, Profile decomposition and metadata. **b** Gene count aggregation pipelines. The pipelines used to build the count matrices are sketched. To build $X^{(AFT)}$, KO, GH and PL are first selected according to the metabolic network in **a**, leading to a list of selected genes (SG) that are annotated in the 9.9M gene catalog and pooled into their respective KO, GH or PL. Some KOs are gathered according to functional proximity, leading to aggregated functional trait (AFT). This aggregation scheme allows to transform sample gene frequencies into AFT frequencies in $X^{(AFT)}$ by pooling SG counts. For prevalent genome (PG) counts, taxonomic marker genes (TMG) are extracted from the genomes with FetchMg and annotated in the 9.9M catalog: the aggregated TMG are next counted in the samples to build $X^{(PG)}$. MGS are reconstructed from the metagenomes, directly counted in the samples and pooled by genus to build $X^{(mgs)}$. A NMF is performed on $X^{(AFT)}$ to obtain $W^{(AFT)}$ (weights) and $H^{(AFT)}$ (functional profiles). Then, nonnegative least square inference is conducted on $X^{(PG)}$ and $X^{(mgs)}$ using $W^{(AFT)}$ as regressor to obtain $H^{(PG)}$ and $H^{(mgs)}$ (PG and MGS taxonomic profiles)

Following the method that was previously used in [25], we assembled a simplified view of the metabolic network of fibre degradation (see Fig. 1a and the “GH, PL and KO Graphical representation” section). Briefly, the first metabolic step was the hydrolysis of fibre, performed by specialized multimodular enzymes belonging to the CAZymes [37, 38]. We performed a two step selection of glycosyl hydrolases (GH) and polysaccharide lyases (PL). We first selected the main GH and PL involved in the catabolism of the main dietary fibre consumed as part of a balanced diet: cellulose, hemicellulose, xylan, resistant starch and pectin [37–43]. Next, since mucin can be used as a substrate by both pathogens and commensals, we included the beta-N-acetyl-glucosaminidase (GH84), fucosidase (GH29 and GH95), neuraminidase/ialidase (GH33) that cleave endogenous mucins and release galactose (GH2), glucose, fucose, or sialic acid moieties [44, 45]. GHs that have a marked fucosidase and galactosidase activity were pooled together for the importance of fucose and galactose pathways. The remaining GHs involved in mucin degradation were gathered and related to sulfate production since mucins are heavily sulfated in the gut and sulfate is accessed during full cleavage of the glycoprotein [46] (Table 2 and Fig. 1A). Polysaccharide lyases PL1, PL9 (pectate lyase) and PL11 (rhamnogalacturonan lyase) were also added. The hydrolysis of fibre and mucin releases oligosides and sugars that are subsequently subjected to anaerobic fermentation. The known fermentation pathways of glucose, fructose, mannose, galactose, L-arabinose, xylose, L-fucose and L-rhamnose were recapitulated using bibliographic ressources [24, 47, 48] and Metacyc database (https://metacyc.org/) guided by expertise [49–53]. We included the Embden-Meyerhoff-Parnas (EMP), Entner-Doudoroff (ED) and semi-phosphorylative Entner-Doudoroff (SP-ED) pathways and the *Bifidobacterium* shunt. The downstream SCFA-producing reactions were added: (i) the three known propionate pathways, including lactate pathways and the propanediol one which is not commonly found in commensals; (ii) butyrate produced from acetate and lactate-utilizing species and (iii) acetate produced through the main pathways but also by some human GI tract pathogens. Finally, H2S, butanediol and acetone production pathways were added, together with the three hydrogen hydrogenotrophic utilization pathways: methanogenesis, sulfate reduction and the Wood-Ljundhal pathway of acetate production from H2/CO2 and glucose (see Fig. 1a). For each pathway, KEGG Orthology (KO) was selected as being representative (KO not involved in other pathway) and essential (the corresponding function is needed for the completion of the pathway) to the given metabolism with the method detailed in [25]. We note that H2S production pathway has been added compared to [25]. See Additional file 12 — Supplementary materials for additional precisions on KO selection.

**Table 2:** KO, GH, PL lists and dataset characteristics. The list of reactions corresponding to Fig. 1 is displayed (top), with their corresponding KO (KEGG nomenclature). Then, GH and PL are listed (bottom)

**Id**	**KO**	
1	K01809	
2	K00882	
3	K06859	
4	K01810	
5	K01803	
6	K00150	
7	K00134	
8	K00927	
9	K00131	
10	K01834	
11	K01689	
12	K00036	
13	K01057	
14	K07404	
15	K01690	
16	K00874	
17	K00041	
18	K01685	
19	K00883	
20	K00849	
21	K00965	
22	K01818	
23	K00879	
24	K01628	
25	K01813	
26	K01804	
27	K01786	
28	K03077	
29	K03080	
30	K00854	
31	K01621	
32	K00169, K00170, K00171, K00172	
33	K00627	
34	K03737	
35	K00656	
36	K04020	
37	K00625	
38	K00925	
39	K00626	
40	K01034, K01035	
41	K00634	
42	K00929	
43	K01574	
44	K01938, K00288, K01491	
45	K00297	
46	K00004, K03366	
47	K00016	
48	K01847	
49	K01848, K01849	
50	K01026	
51	K00672	
52	K01499	
53	K00319	
54	K13942	
55	K00320	
56	K00577, K00578, K00579, K00580, K00581, K00582, K00583, K00584	
57	K00399, K00401, K00402	
58	K01699, K13919, K13920	
59	K13922	
60	K13788	
61	K15024	
62	K00955	
63	K00956, K00957	
64	K00958	
65	K00394, K00395	
66	K11180, K11181	
67	K00380, K00381	
68	K00385	
**GH/PL**		
GH2	GH3	GH5
GH8	GH9	GH10
GH13	GH16	GH26
GH28	GH29	GH30
GH32	GH33	GH35
GH39	GH43	GH44
GH48	GH51	GH74
GH84	GH91	GH94
GH95	GH101	GH115
GH120	GH127	GH129
PL1	PL9	PL11

From the IGC 9.9M genes catalog [5], we extracted the resulting 129,352 selected genes (SG) included in the KO, GH and PL that were further pooled in aggregated functional traits (AFT, see Fig. 1b for a sketch of the selection and aggregation steps). A final list of 101 AFTs characterizing the fibre degradation process in the human gut microbiome was obtained, comprising 33 GH and PL and 68 KOs or KO aggregations (see Table 2 for the complete list of KOs, GHs and PLs that were conserved and the file *List_of_Reactions.xlsx* in the Additional file 11 — Dataset count matrices, Profile decomposition and metadata for the complete list of reactions).

### Metagenomic data and gene frequencies

Gene abundance tables were generated with the METEOR software suite [54]. First, reads were mapped with bowtie2 [55] (parameters: –trim 80 -k 1000) to the integrated gene catalog (IGC) of the human gut microbiome [5], comprising 9.9 million of genes. Alignments with nucleotide identity less than 95% were discarded and gene counts were computed with a two-step procedure previously described that handles multi-mapped reads[30]. Finally, raw gene counts were normalized according to gene length and total number of mapped reads per sample, reported in relative frequency (FPKM normalization).

The IGC KO annotation was used to map the genes to their corresponding AFTs. The GHs and PLs were re-annotated in the IGC using Hmmer [56] and dbCan version 3 [57] with default parameters, after assessment of dbCan annotation quality on 145 manually annotated protein sequences as previously described [25], and the corresponding genes were mapped to their AFTs. The AFT frequencies were obtained by summing the FPKMs of all genes with the corresponding annotation, handling for multiple annotations as previously described [25].

**Fig. 2:**
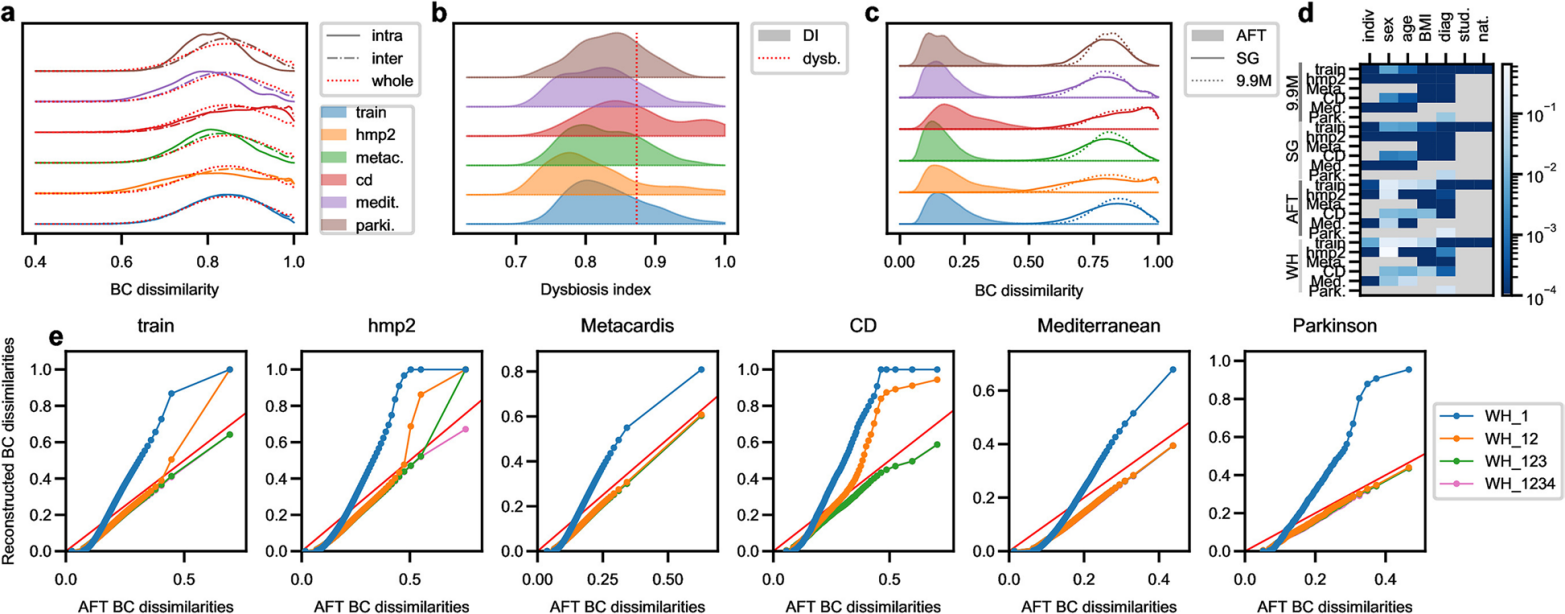
Samples representation with AFT. **a** Intra and inter datasets pBCd distributions are computed on the 9.9M genes for each cohort dataset and compared with pBCd distributions among all samples. Little discrepancies are observed except for the Metacardis and Mediterranean diet cohorts, where intra pBCd is shifted towards lower values, and the CD cohort, where the shift is towards higher values. **b** The dysbiosis index distribution of each dataset is displayed, together with the dysbiosis threshold (red dotted line). Dysbiotic samples are over-represented in the CD cohort. **c** Comparison of different aggregation levels. pBCd distributions are displayed for each dataset, computed both on the 9.9M gene counts, on the subset of SGs or on the AFT counts (see Fig. 1b). pBCd with AFT are strongly decreased. HMP2 and CD distributions are wider than other datasets for all aggregation levels. **d** PERMANOVA *p*-values after variance decomposition analysis of pBCd matrices respectively to structuring co-variables. The PERMANOVA was performed for the different levels of aggregation and for the WH reconstruction. We can see that significance tends to decrease for higher aggregation levels, but the same level of significance is kept between AFT and WH, indicating that the same level of structure is kept after NMF decomposition. **e** Qq-plots of AFT and reconstructed pBCd distributions. The dots indicate the distribution centiles. The reconstructed pBCd are computed on WH reconstructions including 1 $\left(WH_1 = W^{(AFT)}_{1} H^{(AFT)}_1\right)$, 2 $\left(WH_{12} = \sum _{i=1}^2 W^{(AFT)}_{i} H^{(AFT)}_i\right)$, 3 $\left(WH_{123} = \sum _{i=1}^3 W^{(AFT)}_{i} H^{(AFT)}_i\right)$ or the 4 profiles $\left(WH_{1234}=\sum _{i=1}^4 W^{(AFT)}_{i} H^{(AFT)}_i\right)$. The red line indicates the bisector line. We observe that profile 1 alone is not sufficient to reconstruct accurate pBCd but that profiles 1 and 2 together allow the reconstruction of the main part of the pBCd distribution, for the lowest pBCd values. We can see that higher pBCd are not correctly rendered by the 2 profiles, especially for the CD cohort where dysbiotic samples are over represented. Adding the third and the fourth profiles enables a correct reconstruction of the whole distribution but with a homogeneous bias among the whole distribution

At end, a AFT frequency matrix $X^{(AFT)}_{i}$ of dimension $n_{s,i} \times 101$ is built for each dataset $i \in \{train,hmp2,CD,metacardis,med.diet,Parkinson\}$, where $n_{s,i}$ is the number of samples of dataset *i*. The 9.9M genes frequencies are also used to compute pBCd between samples at the three aggregation levels, on the 9.9M genes, on the SGs and on the AFTs as displayed in Fig. 2c (see Fig. 1b for a sketch of the different aggregation levels and the “Statistical treatment” section for methods and Additional file 11 — Dataset count matrices, Profile decomposition and metadata, *X_AFT.xlsx* for the corresponding tables).

### Metatranscriptomic data and frequency matrix

Metatranscriptomic data and metadata were obtained from the HMP2 cohort at https://ibdmdb.org. Gene transcript abundance tables were generated, mapped against the 9.9 million gene catalog and gathered into AFTs with the same procedure as for the metagenomic data. Sample outliers were removed according to read numbers and fraction of reads mapping against the 9.9 catalog, by filtering out respectively the 3 and 5 first centiles. It resulted in the construction of a AFT expression frequency matrix $X^{(AFT,mtx)}$ of dimension $676 \times 101$ (see Additional file 11—Dataset count matrices, Profile decomposition and metadata, *X_AFT.xlsx* for the corresponding table).

### GH, PL and KO Graphical representation

GH and PL were distributed according to the dietary fibre type they degrade. Some GH or PL appear in several arrows because GH or PL CAZymes classification does not represent a unique substrate uptake and fibre degradation modular enzymes are usually not substrate specific. KO were represented by directed arrows linking metabolites together on a graph (Fig. 1a). Note that each array of this graph represent a full metabolic pathway between metabolites, represented by the specific KOs collected for this pathway. Reaction cofactors such as CO_2_, ATP and others were left out of this representation. Extracellular compounds, which micro-organisms can uptake or excrete, were identified with black contours. Functional modules were identified from KEGG and expert knowledge. The metabolic network has been displayed with Pathvisio [58] (Fig. 1a) and further annotated (functional blocks) with Inkscape [59].

### Prevalent genome selection and function frequencies computation in prevalent genomes

A list of 203 genomes (see Additional file 11—Dataset count matrices, Profile decomposition and metadata, *Genome_list.xlsx*) was built by selecting prevalent genomes from [27] and [60], taking care that the main phyla are represented. The genes involved in the 101 AFTs were recovered in 191 genomes (see Additional file 11—Dataset count matrices, Profile decomposition and metadata, *Genome_list.xlsx* for subset list): KEGG Orthology annotation was carried out using diamond (0.7.11) [61] and default parameters on the KEGG database from 2016 [62]. If a query was found to have multiple hits, only the best hit was kept, any hit with bitscore under 60 was discarded [29]. GH and PL annotations were obtained using Hmmer [56] and dbCan version 3 [57] with default parameters. The resulting presence/absence annotation is given in (see Additional file 11—Dataset count matrices, Profile decomposition and metadata, *Genome_list.xlsx*) and used for clustering in Fig. S6 (see the “Statistical treatment” section).

### Taxonomic count matrices

Two different taxonomic informations were derived by counting in the samples either the 203 PGs through annotation of taxonomic marker genes or metagenomic species. 40 taxonomic marker genes (TMG) [63–65] were extracted from each 203 gut microbiota PGs using fetchMG (http://vm-lux.embl.de/~mende/fetchMG/about.html) [66] with default parameters. These genes were annotated in the IGC catalog using diamond (0.7.11) [61] and default parameters. Any hit with bitscore, percent identity or alignment length under respectively 60, 97 and 45 was discarded as indicated in [66] for correct taxonomic annotation. TMGs frequencies in each sample were pooled by PG to assemble a genome frequency matrix $X^{(PG)}$ (see Additional file 11—Dataset count matrices, Profile decomposition and metadata, *X_PG.xlsx*). Metagenomic species (MGS) [18] were recovered in the *train* dataset. Genus abundance was computed according to MGS abundance in order to assemble a MGS-derived genus frequency matrix $X^{(mgs)}$ (see Additional file 11—Dataset count matrices, Profile decomposition and metadata, *X_mgs.xlsx*).

### Inference of functional profiles

The inference method was thoroughly detailed in [25]. Briefly, starting from the frequence matrix $X^{(AFT)}_{train}$ of the 101 AFTs of the *train* dataset, we used a constrained nonnegative matrix factorization (NMF) to decompose $X^{(AFT)}_{train}$ as the product of two nonnegative matrices, the profile matrix $H^{(AFT)}$ of dimension $k \times 101$ and the weight matrix $W^{(AFT)}_{train}$ of dimension $n_{s,train}\times k$ where *k* is the number of profiles, an hyperparameter to be tuned (see below). Each line of $H^{(AFT)}$ represents a functional profile, characterized by a vector of co-varying AFT frequencies: $H^{(AFT)}_{i,j}$ is the frequency of AFT *j* in profile *i*. The columns of $W^{(AFT)}_{train}$ represent the weights of the corresponding profiles in the different samples: $W^{(AFT)}_{train\quad i,j}$ represents the weight of profile *j* in the *i*-th sample of the *train* dataset $X^{(AFT)}_{train}$.

Matrices $W^{(AFT)}_{train}$ and $H^{(AFT)}$ are inferred by solving the optimization problem

1 $$\begin{aligned} \left(W^{(AFT)}_{train},H^{(AFT)}\right) = \underset{\begin{array}{c} W\ge 0 \\ H \ge 0 \\ FH^T \le 0 \end{array}}{\text {arg}\,\text{min}} \left\| \left(X^{(AFT)}_{train} - WH\right)D^{-1} \right\| ^2_F + \alpha \left( \Vert W\Vert ^2_F + \left\| H D^{-1} \right\| ^2_{1,2} \right) \end{aligned}$$

In this equation, *D* is a diagonal scaling matrix, so that $D_{ii} = \left\| X^{(AFT)}_{train\quad i} \right\|_2$ is the $l_2$ norm of the *i*-th column. The matrix *F* is a constraint matrix designed to favour the presence in the profiles of complete metabolic pathways linking two extracellular compounds in Fig. 1a so that a given profile carries the whole set of reactions needed for intracellular metabolism (see Additional file 12—Supplementary materials for additional precisions on the construction of *F*, Additional file 11—Dataset count matrices, Profile decomposition and metadata, *F.xlsx* for the constraint matrix and [25] for more details). Finally, 155 constraints were implemented so that *F* has dimension $155 \times 101$. The parameter $\alpha$ is a tuning parameter that sets up the impact of the regularization penalties $\Vert W\Vert ^2_F + \Vert H D^{-1} \Vert ^2_{1,2}$ on the NMF. The Froebenius norm in penalty $\Vert W\Vert ^2_F$ tends to standardize the Profile weights in a given sample while the $l_{1,2}$ norm on *H* tends to assign each AFT to a limited number of profiles by inducing sparsity on the rows of *H*. The resulting profiles are not exclusive, meaning that a given AFT can be represented in several profiles.

The selection of the regularization parameter $\alpha$ and the number of profiles *k* was performed using the same triple criterion approach as in [25] providing the best trade-off between internal data reconstruction (reconstruction error criterion), reconstruction of external samples (bi-cross validation) and profiles stability, while avoiding over-fitting. See Additional file 12—Supplementary materials for precise definitions of the hyperparameter selection criteria.

Implementation of the NMF inference in python based on OSQP solver [67] is available at https://forgemia.inra.fr/nmf4metagenomics/pynmf and is based on a block coordinate descent algorithm consisting in alternatively solving the nonnegative least-square problems inferring $W^{(AFT)}_{train}$ knowing $H^{(AFT)}$ with

2 $$\begin{aligned} W^{(AFT)}_{train} = \underset{\begin{array}{c} W\ge 0 \end{array}}{\text {arg}\,\text{min}} \left\|\left(X^{(AFT)}_{train} - W H^{(AFT)}\right)D^{-1} \right\| ^2_F + \alpha \left( \Vert W\Vert ^2_F \right) \end{aligned}$$

and inferring $H^{(AFT)}$ knowing $W^{(AFT)}_{train}$

3 $$\begin{aligned} H^{(AFT)} = \underset{\begin{array}{c} H \ge 0 \\ FH^T \le 0 \end{array}}{\text{arg}\,\text{min}} \left\| \left(X^{(AFT)}_{train} - W^{(AFT)}_{train}H\right)D^{-1} \right\| ^2_F + \alpha \left( \left\| H D^{-1} \right\| ^2_{1,2} \right) . \end{aligned}$$

Average profiles weights $\bar{W}^{(AFT)}_{train}$ and AFT counts $\bar{X}^{(AFT)}_{train}$ of the training set are defined. Namely, average profiles weights $\bar{W}^{(AFT)}_{train}=\frac{1}{n_s} \sum _{i=1}^{n_s} W^{(AFT)}_{train,i}$ are computed by averaging W on the train set. Average AFT counts $\bar{X}^{(AFT)}_{train}=\frac{1}{n_s} \sum _{i=1}^{n_s} X^{(AFT)}_{train,i}$ are obtained in the same manner.

### Profiles validation

The matrix $H^{(AFT)}$ whose lines are the 4 functional Profiles obtained after NMF on $X^{(AFT)}_{train}$ was held fixed, and the positive least square regression (2) was performed on the validation datasets $X^{(AFT)}_{d}$, for $d \in \{hmp2,CD,metacardis,med.diet,Parkinson\}$ to determine the corresponding weight matrices $W^{(AFT)}_{d}$. Relative reconstruction error distributions $\left\| {X^{(AFT)}_{d}}_{i} - {W^{(AFT)}_{d}}_{i} H^{(AFT)}\right\| _F\left/\left\| {X^{(AFT)}_{d}}_{i}\right\|_{F}\right.$ for $i=1,\dots ,n_{s,d}$ are computed for validation assessment.

### Genomes and MGS affectation to profiles

To affect genomes to the functional profiles, we assumed that the weights predicting profiles assemblage to reconstruct $X^{(AFT)}_{train}$ were also a suitable predictor to reconstruct genome frequencies. In other words, we search for genomes that co-vary with the functional profiles, with the implicit assumption that the genes included in a functional profile will vary proportionally with the genomes that carry them. Hence, knowing the $(1153 \times 4)$ matrix $W^{(AFT)}_{train}$, the unconstrained positive least square regression (3) was solved on respectively the prevalent genomes and the MGS frequency matrices $X^{(PG)}_{train}$ and $X^{(mgs)}_{train}$ to infer $H^{(PG)}$ the $4 \times 203$ prevalent genome and $H^{(mgs)}$ the $4 \times 217$ MGS-derived genus count matrices. Note that the same $L_{1,2}$ regularization penalty as in Eq. (1) was applied to favour unique allocation to the profiles, together with the same penalty coefficient $\alpha$.

To compare this profile taxonomic make up with taxonomic-only profiling of the metagenomes, we performed a NMF on the taxonomic count matrix $X^{(PG)}$ and $X^{(mgs)}$. Namely, we solved problem (1) on $X^{(PG)}_{train}$ and $X^{(mgs)}_{train}$ to find respectively the couples $\left(W^{(PG,nmf)}_{train},H^{(PG,nmf)}\right)$ and $\left(W^{(mgs,nmf)}_{train},H^{(mgs,nmf)}\right)$ that best approximate the count matrices. Note that the constraint $FH^T \le 0$ has been removed and that the regularization parameter $\alpha$ that is used is the same than for problem (1).

### Transcriptome affectation to profiles

We use the same methodology as for taxonomic affectation: we search for transcripts co-varying with the functional profiles. We then infer $H^{(AFT,mtx)}$ such that $W^{(AFT)}_{hmp2} H^{(AFT,mtx)} \simeq X^{(AFT,mtx)}$ where $W^{(AFT)}_{hmp2}$ is the weight matrix obtained by decomposition of the metagenomic data with the function profiles in the *hmp*2 dataset. The matrix $H^{(AFT,mtx)}$ is obtained with the non-negative least square inference problem (3) with the same constraint matrix *F*, $L_{1,2}$ regularization penalty and penalty coefficient.

### Statistical treatment

All the computations and statistics have been performed with custom scripts using the standard python libraries numpy [68], scipy [69], pandas [70] and matplotlib [71]. Ternary plots that are plots in barycentric coordinates of normalized $W_1$, $W_2$, and $W_3$ values, i.e. $W_i/(W_1+W_2+W_3)$ for $i=1,2,3$, are produced with the Ternary python package [72] (Figs. 6a, c and e, 7, S4a, c and e, S7c and d).

pBCd have been computed with scikit-learn [73] (see Fig. 2). Intra-cohort pBCd refers to dissimilarities obtained with two samples of the same cohort while inter-cohort pBCd distribution of the dataset $i \in \{train,hmp2,CD,metacardis,med.diet,Parkinson\}$ refers to dissimilarities obtained with a sample from the dataset *i* and another sample from dataset $j \ne i$.

Dysbiosis index (DI) has been computed following [4]: a reference set has been set up with non-IBD samples of the *‘hmp2’* cohort obtained after the 20-th weeks from the patient enrollment and DI is defined as the median pBCd with the reference dataset, excluding samples from the same individual. A dysbiotic threshold is defined as the quantile 0.9 of the DI in healthy samples: samples with DI above this threshold are tagged as dysbiotic [4].

To avoid statistical bias (individual effect) due to over-representations of the same individuals, only the first time point of each individual is included in differential analysis involving the ‘hmp2’ cohort, i.e. for BMI (Fig. 5a, b), CD and dysbiosis analysis (Fig. 6a–d).

PERMANOVA (Fig. 2d) has been performed on the intra-cohort pBCd matrices obtained from the different levels of aggregation (9.9M genes, SGs and AFTs, see the “A functional view of fibre degradation in metagenomes” section) with scikit-bio [74] using 10000 permutations and default parameters, respectively to the following structuring co-variables: individual, sex, age, body mass index (BMI), diagnosis, study and nationality.

All the statistical tests have been performed with the scipy.stats module [69] (Fig. 5, Fig. 6, and Fig. S4). Multiple test corrections were made with statsmodels.stats.multitest [75] (Fig. 7 and Fig. S5). In all graphs, significant *p*-values are indicated with one star if $1e-2<p\le 5e-2$, 2 stars if $1e-3<p\le 1e-2$, 3 stars if $1e-4<p\le 5e-3$ and 4 stars if $p\le 1e-4$, non significant *p*-values are indicated with *n.s.*. The test name is indicated with the significance level. MW stands for the ‘two-sided’ Mann-Whitney *U* test and levene for the Levene test for the variance.

Support vector machine (SVM) classification has been made with scikit-learn [73] using ‘rbf’ kernel after cross-validation of the hyperparameters *C* and $\gamma$ and min-max scaling normalization. The SVM classifier was trained on the ‘hmp2’ cohort, by classifying CD against healthy samples (Fig. S5).

Hierarchical clustering has been performed with the package scipy.cluster.hierarchy using a pairwise Jaccard distance matrix computed on the AFT presence-absence in the 191 genomes and the 4 profiles (see the “Prevalent genome selection and function frequencies computation in prevalent genomes” section), ward algorithm and 4 clusters (Fig. S6).

## Results

### Assessment of dataset and gene selection

Upstream to any data analysis, we first assess that the training set is representative of the whole set of metagenomes included in the study by computing pBCd on the 9.9M genes, focusing on intra and inter-cohort distributions (see the “Statistical treatment” section). The training set shows nearly identical intra- and inter-cohort pBCd distributions that are also very close to the pBCd distribution obtained when the whole set of sample pairs are pooled (Fig. 2a, dashed and plain blue curves superimposed with dotted red curve), indicating that the training set is representative of the gene diversity observed in the metagenomes of the different datasets. The intra- and inter-cohort pBCd of the CD cohort show a pick of high dissimilarities (Fig. 2a, red curve), showing a higher prevalence of dissimilar samples in agreement with the over-representation of dysbiotic samples in this cohort (Fig. 2b, red). A similar observation can be done for the hmp2 cohort, with slighter effects, that can be related to the over-representation of inflammatory bowel diseases (IBD) in these cohorts. On the contrary, the Mediterranean diet cohort presents a higher fraction of samples with low dysbiosis index (Fig. 2b, purple).

We next check that the functional simplification operated in this study by selecting genes related to fibre degradation does not strongly bias the functional variability observed in the metagenome. Indeed, as fibres are the main substrate in the gut, fibre-related pathways are expected to be observed in all the metagenomes, inducing less variable counts that could impair sample differentiation. We then assess the impact of the different levels of aggregation and simplification of the metagenome performed in the study (see Fig. 1b and the “A functional view of fibre degradation in metagenomes” section). The pBCd obtained on the selected genes (SG) frequencies (Fig. 2c, plain lines) show very similar distributions to the pBCd computed on the 9.9M genes (Fig. 2c, dotted lines), indicating that the functional simplification resulting from the gene selection allows to reproduce the same sample stratification as the one obtained from the whole metagenome. As expected, dissimilarities are strongly reduced when pooling the SGs in AFTs shifting pBCd towards lower values (Fig. 2c, colored distributions), but AFT-based pBCd captures the over-representation of dissimilar samples in the CD and hmp2 cohorts. Furthermore, PERMANOVA shows that the main part of dataset structures with respect to co-variables are correctly reproduced by AFT-based pBCd (Fig. 2d), indicating that the functions related to fibre degradation selected for the AFTs are suitable to capture stratifications observed in the whole metagenome.

### Fibre degradation process is accurately described by 4 universal functional profiles

#### Statistical inference of the functional profiles

Co-varying AFTs are identified in the training dataset using the NMF method (see the “Inference of functional profiles” section), resulting in 4 distinct functional profiles (matrix $H^{(AFT)}$) whose weighted mixture with weights $W^{(AFT)}_{train}$ allows to reconstruct the training AFT counts $X^{(AFT)}_{train}$: $X^{(AFT)}_{train} \simeq W^{(AFT)}_{train} H$ (mean relative error : 17%, see Fig. S1a). We recall that the NMF method was specifically constrained by a metabolic-based constraint *F* favouring in practice the clustering in the same profile of AFTs belonging to the same metabolic pathways [25]. This constraint results in the distribution of the different metabolic pathways of the fibre degradation network among the 4 profiles.

#### Validation on external datasets

To assess the ability of the profiles to reconstruct external datasets, i.e. to validate the universality of the functional profiles, the nonnegative least square regressions (2) is performed on the AFT count matrix $X^{(AFT)}_{d}$ in order to identify the best weight matrix $W^{(AFT)}_{d}$ so that $X^{(AFT)}_{d} \simeq W^{(AFT)}_{d} H^{(AFT)}$ with $d \in \{hmp2,CD,metacardis,med.diet,Parkinson\}$. The relative reconstruction error distributions are very homogeneous across datasets, except for the CD dataset where increased reconstruction errors are observed (Fig. S1a). This is probably induced by an over-representation of dysbiotic and CD samples in this dataset that are less acurately reconstructed (Fig. S1 d and g). Structuring variables such as study, health or weight status, drug administration, diet or dysbiosis do not strongly affect reconstructions (Fig. S1). In the worst case (dysbiotic samples), the mean relative error is kept under 27%, and the 0.95 quantile is kept under a relative error of 44%.

We note a strong discrepancy in the four profile weights in the samples (Fig. S1 j). The weights $W_1$ and $W_2$ of profiles 1 and 2 are significantly higher than $W_3$ and $W_4$ in all datasets (paired *t*-test, p$<1e-6$). This observation suggests that profiles 1 and 2 carry characteristic gut microbiota fibre degradation functions dominant in the majority of metagenomes whereas profiles 3 and 4 indicate specific functional variations.

To investigate the contribution of the different profiles to metagenome reconstruction, we compare the pBCd obtained on reconstructed counts with AFT-based pBCd when the number of profiles is increased. Namely, we compute the reconstructed count matrices $\sum _{m=1}^K W^{(AFT)}_{d,m} H^{(AFT)}_m$ for $K=1$ to 4 and compared the resulting AFT-based pBCd with the AFT-based pBCd computed on the original count matrix $X^{(AFT)}_{d}$, for $d\in \{hmp2,CD,metacardis,med.diet,Parkinson\}$ (Fig. 2. e). We can see that the first profile alone does not provide an accurate reconstruction of the pBCd distribution. Interestingly, adding the second profile allows to reconstruct the main part of the pBCd distributions (until approximatively the 80th centile in the worst case, Fig. 2e, CD, orange line), except when the dataset involves over-representation of highly dissimilar samples (HMP2 and CD datasets, Fig. 2c, orange and red distributions). However, in these cases, adding the third profile (and even the fourth for the *hmp2* dataset, Fig. 2e, HMP2, green and pink lines) makes it possible to reconstruct higher pBCds. These observations suggest that profiles 1 and 2 carry sufficient information to describe the AFT-related metagenomic variability in the main part of the population, except in dysbiotic situations, that are correctly rendered by adding profiles 3 and 4 in the reconstruction. We also note that the reconstructed pBCds are slightly uniformly underestimated, the qq-plot lying slightly under the bisector line.

### The four profiles present contrasted functional characteristics

**Fig. 3:**
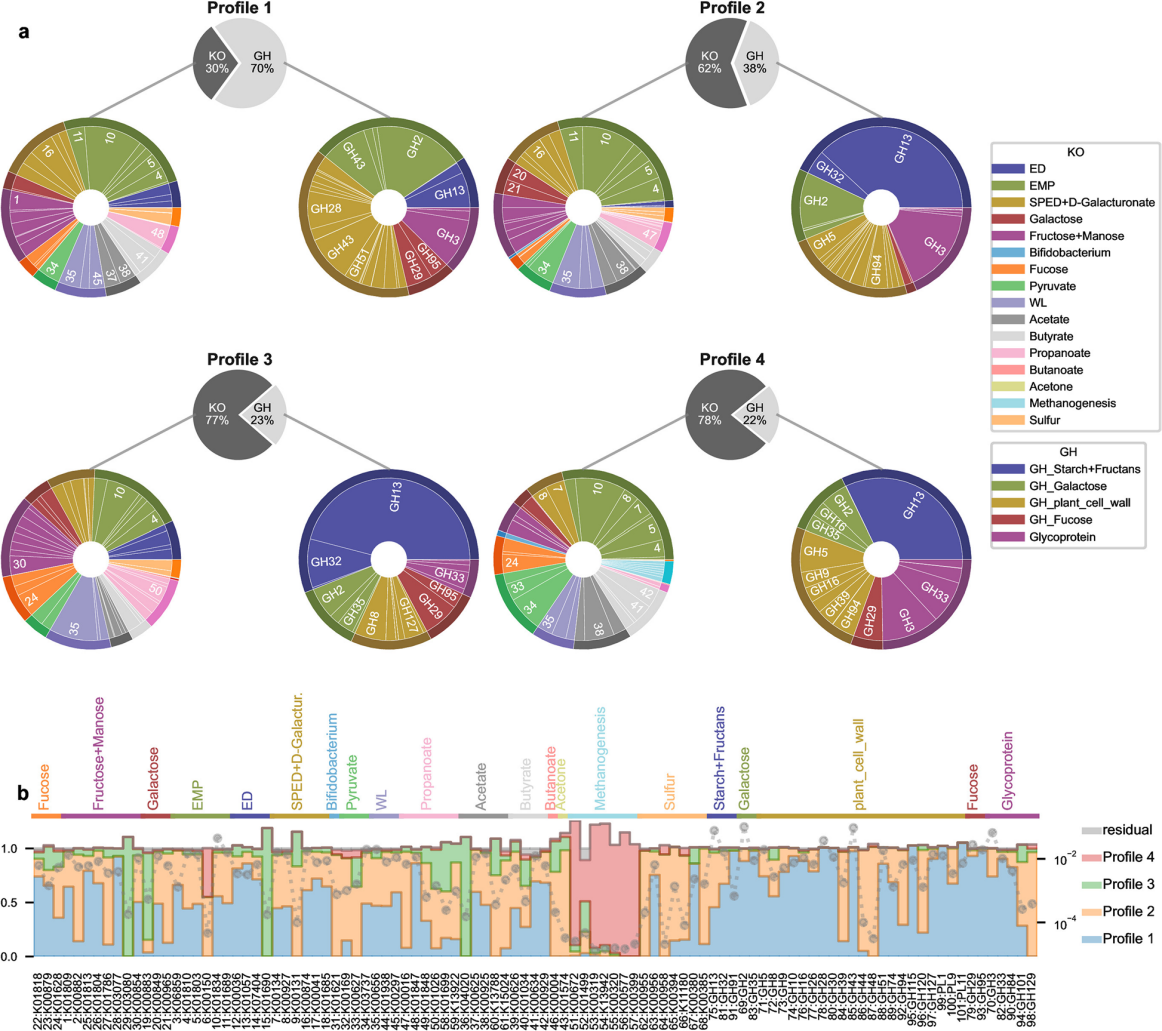
Functional profiles characterization. **a** KO and GH-related AFT frequencies are first gathered to show the distribution of KO and GH in each profile (top central pie chart). Then, the frequency of each AFT is renormalized by KO or GH/PL total frequency, and displayed in pie-charts for KO (left) and GH/PL (right) after clustering by functional modules (color coded; see Fig. 1a for the functional modules). The number of the KO or GH-related AFT is displayed in its corresponding pie-chart sector (radially, inner zone) when its frequency is higher than 3% in the profile. **b** Average profile contribution in AFT counts. Average profile contribution for AFT *j* and profile *i* is computed as the proportion of average AFT counts provided by the profile *i* with $\bar{W}^{(AFT)}_{train,i} H^{(AFT)}_{ij} / \bar{X}^{(AFT)}_{train,j}$, where $\bar{W}^{(AFT)}_{train}$ and $\bar{X}^{(AFT)}_{train}$ are introduced in the “Inference of functional profiles” section. Finally, contributions are stacked by AFT in bar plots and ordered by functional modules. The residual $1-\sum _{i=1}^4\bar{W}^{(AFT)}_{train,i} H^{(AFT)}_{ij} / \bar{X}^{(AFT)}_{train,j}$ is plotted in gray. Dotted gray lines indicate the value of $\bar{X}^{(AFT)}_{train,j}$ measuring the average AFT frequency (*y* log-scale on the right)

To dig into the intrinsic functional characteristics of the different profiles, we plot their AFTs distributions (Fig. 3a and Additional file 10—metabolic exploration). We first observe that the different profiles do not exhibit the same balance between GHs, i.e. AFTs involved in complex molecule cleavage like fibres, and KOs, i.e. AFTs taking in charge the downstream part of fibre degradation, from simple sugars to end products (Fig. 1a). Profile 1 carries the largest set of GH (70%), reflecting a very broad capacity to breakdown fibre, resistant starch and diverse plant cell wall polymers, unlike profile 2 (38%), profile 3 (23%) and profile 4 (22%). Profile 1 main GHs are related to mucin (GH2, GH43, GH29, GH95), glycoprotein and xylan (GH3), pectin and plant cell wall (GH 43, GH28), and to a less extent to starch degradation (GH13) as shown in Fig. 3a (GH pie chart) and Additional file 10—metabolic exploration. Profiles 2, 3 and 4 are shifted towards sugar fermentation rather than hydrolysis. They are preponderantly characterized by starch degradation and amylase (GH13), with secondary GH activity related to glycoprotein and xylan degradation (GH3) and mucin (GH2) for profile 2, fructan and inulin degradation (GH32) for profile 3 and cellulose degradation (GH5) for profile 4. Profiles 2 and 4 present high proportions of GH involved in glycoprotein degradation. In contrast, profile 3 has noticeably low proportions of GH involved in plant cell wall breakdown compared to other profiles but presents high proportions of GH2 releasing galactose from N acetyl-galactosamine moieties and GH29 and GH95 releasing fucose, suggesting a shift from polymers hydrolysis towards host derived glycan breakdown.

In the downstream part of fibre degradation, profile 1 and profile 2 are very similar (Fig. 3a, KO pie charts and Additional file 10—metabolic exploration). The main differences are related to galactose pathway (AFT 21 is more present in profile 2) and in the propanoate pathway where profile 1 takes in charge AFT 48 linking lactate to propanoate while profile 2 is involved upstream in AFT 47 linking pyruvate to lactate. Profiles 3 and 4 present more dissimilarities: EMP proportion is reduced in profile 3 while fucose (AFTs 22, 23 and 24) and propanoate (AFT 48 and 50) pathways are enhanced (Fig. 3a and Additional file 10—metabolic exploration). Profile 3 is also the unique profile providing AFT 19 in galactose pathway. Profile 4 is characterized by a higher proportion of AFTs of the pyruvate pathway and the presence of the methanogenesis.

To check if these profiles, which represent a functional potential, are expressed in a metatranscriptome, we assembled a AFT transcript count matrix $X^{(AFT,mtx)}$ obtained from the *hmp*2 database and searched for co-varying AFT transcripts with the profiles. Namely, we performed a nonnegative inference to define the expression profile matrix $H^{(AFT,mtx)}$ so that $X^{(AFT,mtx)}\simeq W^{(AFT)}_{hmp2}H^{(AFT,mtx)}$ (see Fig. S8a, b and c for approximation accuracy). The resulting AFT distribution of these expression profiles are represented in Fig. S8d. The expression profiles 1 and 2 shows a very good agreement with the functional profiles 1 and 2 that are the preponderant profiles of the metagenome (Figs. S8d and Fig. 3a, upper panels). This consistency is particularly striking since the expression of a gene is not directly related to its count in the metagenome: it may reflect the preponderance of fibre degradation in the gut. The expression profile 3 gathers the same main AFTs than profile 3 but with different weights. In particular, the relative expression of EMP and WL pathways and of GH32 is higher than their functional potential in the metagenome. The expression profile 4 only gathers methanogenesis-related AFT transcripts, confirming the functional characterization of profile 4.

### Profile contribution to the microbiota functional potential

These intrinsic characteristics functionally characterize each profile but do not give insight into its importance in the metagenomes. We assess the relative contribution of each profile *i* to the total count of AFT *j* by computing $\bar{W}^{(AFT)}_{train,i} H^{(AFT)}_{ij} \left/ \bar{X}^{(AFT)}_{train,j}\right.$, where $\bar{W}^{(AFT)}_{train}$ and $\bar{X}^{(AFT)}_{train}$ are the average weights and counts in the training set as defined in the “Inference of functional profiles” section. The four profiles have different ecological contributions in the metagenomes (Fig. 3b and Fig. S2a). As expected, profile 1 is the main provider of GH counts, except for GH with the lowest counts (GH44 and 48 for plant cell wall degradation, GH 101 and 129 for glycoprotein cleavage). It is also particularly involved in some pathways such as *Bifidobacterium* shunt, butyrate production, WL, SPED, EMP, ED, fructose and fucose pathways. Profile 2 has a major contribution in the pyruvate, butanoate, acetone pathway and some specific KOs (K00882 and K01786 in the fructose pathway, K00965 for galactose metabolism, K13788 for acetate pathway). Profile 3 is the unique provider of some KOs such as K03080 in the fructose pathway, K01690 in ED or K04020 in acetate production. It is also particularly present in galactose, fucose, SPED and propionate production. Profile 4 is the main contributor for methanogenesis, and has otherwise small to marginal contributions in EMP, pyruvate or sulfur pathways.

### Taxonomic make up of the 4 profiles

A natural question at this point is to wonder which taxonomic units could provide the AFT of each functional profiles. We selected 203 genomes among the top-prevalent strains in metagenomes, covering the main phyla found in the gut microbiota (see the “Prevalent genome selection and function frequencies computation in prevalent genomes” section) and assembled TMG count matrix $X^{(PG)}$ for the different metagenomic datasets (see the “Taxonomic count matrices” section). Under the assumption that a genome providing a specific AFT in a functional profile $H^{(AFT)}_i$, $i=1,\cdots ,4$, should co-vary with the profile, we search by nonnegative inference the best $H^{(PG)}$ so that $X^{(PG)}_{train} \simeq W^{(AFT)}_{train} H^{(PG)}$. In this equation, $W^{(AFT)}_{train}$ is the weight matrix of the functional profiles (see Fig. 1b the “Genomes and MGS affectation to profiles” section). Hence, if $H^{(PG)}$ is consistent, we should also have for each external dataset $d \in \{hmp2,CD,metacardis,med.diet,Parkinson\}$ $X^{(PG)}_{d} \simeq W^{(AFT)}_{d} H^{(PG)}$. This is actually the case since the reconstruction errors at the phyla levels (Fig. S3) follow similar characteristics to the reconstruction of the AFT counts (Fig. S1). The same inference procedure is performed to reconstruct the training MGS count matrix $X^{(mgs)}_{train}$ resulting in the MGS profile matrix $H^{(mgs)}$ with similar reconstruction error distributions (Fig. S3j).

### Marked taxonomic structure of the profiles

**Fig. 4:**
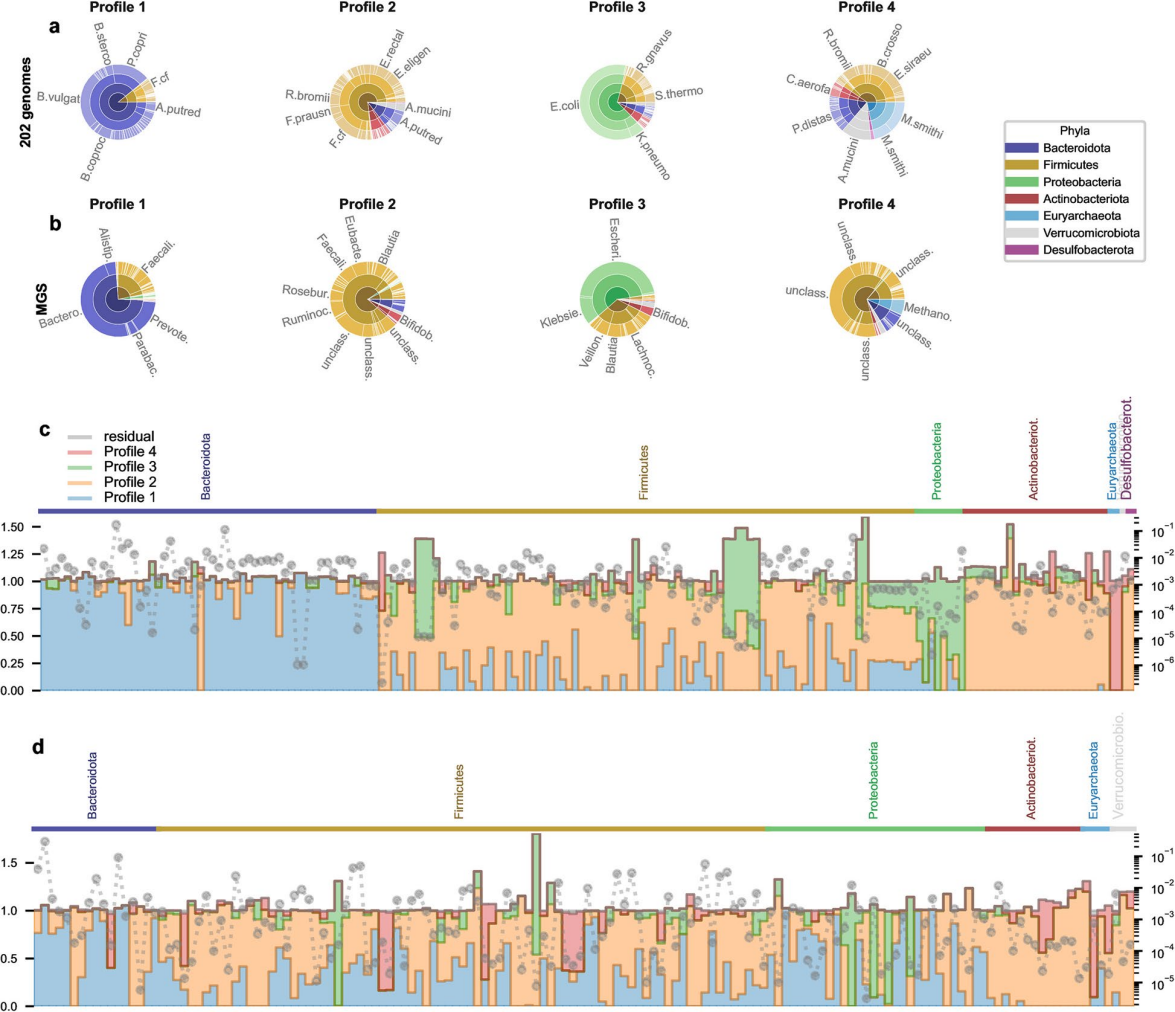
Taxonomic profiles characterization. **a** The 203 genomes frequencies in $H^{(PG)}$ are displayed in pie-charts and clustered by successive taxonomic levels, i.e. taxa (outer ring), genus, class and phyla (inner ring), color-coded by phyla. Taxa names are displayed radially when their frequency is higher than 1% in the profile. These taxa are recapitulated in Table 3. **b** The same procedure is applied on MGS clustered at the genus level. Taxonomic levels are genus, class and phyla. **c** Average profile contribution in the 203 genomes counts. Namely, the same average profile weight $\bar{W}^{(PG)}_{train}$ as in Fig. 3 is computed together with $\bar{X}^{(PG)}_{train}$. Then, average profile contribution for genome *j* and profile *i* is computed with $\bar{W}^{(PG)}_{train,i} H^{(PG)}_{ij} \left/ \bar{X}^{(PG)}_{train,j}\right.$. Finally, contributions are stacked by genome in bar plots and ordered by phyla. The residual $1-\sum _{i=1}^4 \bar{W}^{(PG)}_{train,i} H^{(PG)}_{ij} \left/ \bar{X}^{(PG)}_{train,j}\right.$ is plotted in gray. Dotted gray lines indicate the value of $\bar{X}^{(PG)}_{train,j}$ measuring the average AFT frequency (*y* log-scale on the right). **d**) The same procedure is repeated on the MGS clustered at the genus level

The taxonomic profiling obtained with the MGS or the 203 PGs are particularly consistent (Fig. 4a, b). Profile 1 is dominated by *Bacteroidetes* species belonging to the genera *Bacteroides* and *Prevotella*. In contrast, Profile 2 has a high diversity of *Firmicutes* species, with butyrate-producing species from the Cluster IV *Faecalibacterium prausnitzii* species, *Roseburia intestinalis*, *Ruminococcus bromii* which is a degrader of resistant starch [76], and cluster XIVa *Eubacterium rectale* such as *Eubacterium eligens*. *Anaerostipes putredinis* is the main representer of the *Bacteroidetes* phylum. *Actinobacteria*, including the *Bifidobacteria* and the *Verromicrobia* species *Akkermansia muciniphila* are also present in profile 2. Profile 3 is strikingly distinct from the two first profiles. It has a major proportion of commensals of the *Proteobacteria* phylum (*Escherichia coli* K12 and *Klebsiella pneumoniae*) but also marginaly the multi-drug resistant *Escherichia coli* SMS-3-5 strain [77] and *Citrobacter sp*. The mucin degrader *Ruminococcus gnavus* [78] is the main representer of the *Firmicutes*. Within the *Bacteroidetes*, the main fibre hydrolysing species are not contributing but the *Bacteroides fragilis* are dominant. *Bifidobacteria* and *Akkermansia muciniphila* are also part of Profile 3 taxonomic contribution but more marginally. Profile 4 is significantly distinct regarding its taxonomic representation. The *Euryarchaeota* domain, and specifically with hydrogenotrophic methanogenic strains from *Methanobrevibacter smithii* species [79], is over-represented. Then, follow *Firmicutes*, *Verrucomicrobia* (*Akkermansia Muciniphila*), *Bacteroidetes* and *Actinoacteria* (see Table 3 for a table of main PGs in profiles). The MGS profiling of profile 4 is rather different: it also includes the methanogens but otherwise gathers unclassified genus. These discrepancies can be related to the low amount of signal carried by profile 4 (Fig. S1j).

**Table 3:** For each profiles, PGs with a frequency greater than 1% are indicated

Profile 1	Profile 2	Profile 3	Profile 4
*Prevotella copri*	*Eubacterium eligens*	*Streptococcus thermophilus*	*Eubacterium siraeum*
*Bacteroides stercoris*	*Eubacterium rectale*	*Ruminococcus gnavus*	*Butyrivibrio crossotus*
*Bacteroides vulgatus*	*Ruminococcus bromii*	*Escherichia coli*	*Ruminococcus bromii*
*Bacteroides coprocola*	*Faecalibacterium prausnitzii*	*Klebsiella pneumoniae*	*Collinsella aerofaciens*
*Alistipes putredinis*	*Faecalibacterium cf*		*Parabacteroides distasonis*
*Faecalibacterium cf*	*Alistipes putredinis*		*Akkermansia muciniphila*
	*Akkermansia muciniphila*		*Methanobrevibacter smithii*

We now wonder how consistent are the profiles with the enterotypes obtained from the analysis of the taxonomic compositions of large metagenomic datasets [20, 33]. Profiles 1 and 2 present contrasted distribution among enterotypes (Fig. S4 c and d): if profile 1 is over-represented in Bact2 and Prevotela enterotypes, higher weights $W^{(AFT)}_{2}$ are observed for Bact1 and Ruminoccocus enterotypes. Interestingly, profile 3 is almost only observed in Bact2 enterotypes and profile 4 in Ruminoccocus enterotype (Fig. S4 d).

### The profiles link the taxonomic and functional composition of the microbiota

Compared to the functional contribution of the profiles (Fig. 3b), their taxonomic contribution is very structured (Fig. 4c, d and Fig. S2b, c). Profile 1 is the main contributor for *Bacteroidetes*, profile 2 for *Firmicutes* and *Actinobacteriota*, profile 3 for the *Proteobacteria* and some *Firmicutes* and profile 4 for the *Euryarchaeota*. Repeating this analysis on MGS clustered by genus (Fig. 4d and Fig. S2c) leads to consistent results, despite the very different nature of the taxonomic data, i.e. targeted PGs versus untargeted MGS. This clear structure is particularly strinking since the taxonomic profiling is indirect and based on the profiles weights obtained on the AFT counts, indicating that these specific phyla may carry specific AFTs of the different profiles, linking taxonomic composition and functional contribution to the metagenome.

To check this hypothesis, we blasted the genes involved in the AFTs in 191 PGs (Fig. S6) and clustered the genomes by their similarity in carrying AFT genes, adding the four profiles to the clustering process (see the “Statistical treatment” section). The *Bacteroidota*, main carrier of GH genes, clustered with profile 1 as expected. *Actinobacteria* clustered together, characterized by the *Bifidobacterium* shunt and one function involved in acetate production (AFT 60). *Firmicutes* are splitted in two groups: the first group characterized by the absence of fucose-related genes and little presence of fructose and mannose pathways clustered with profile 4, while the others clustered with profiles 2 and 3. Profile 3 clustered with the *Proteobacteria* characterized by a strong representation of fucose, fructose, mannose and propionate pathways. This clustering is very consistent with the taxonomic profiling, even though derived from very different biological signals. This repeated consistency (profiling with targeted PGs, untargeted MGS, clustering based on AFT presence/absence in genomes) suggests that the functional stratification described by the different profiles actually reflects co-variations of microbial entities. These covarying taxons, characterized by within-group functional similarities and between-group functional discrepancies, are the taxonomic support of the covarying AFTs defining the functional profiles.

### Balance of profiles 1 and 2 reflects metabolic status and dysbiosis

**Fig. 5:**
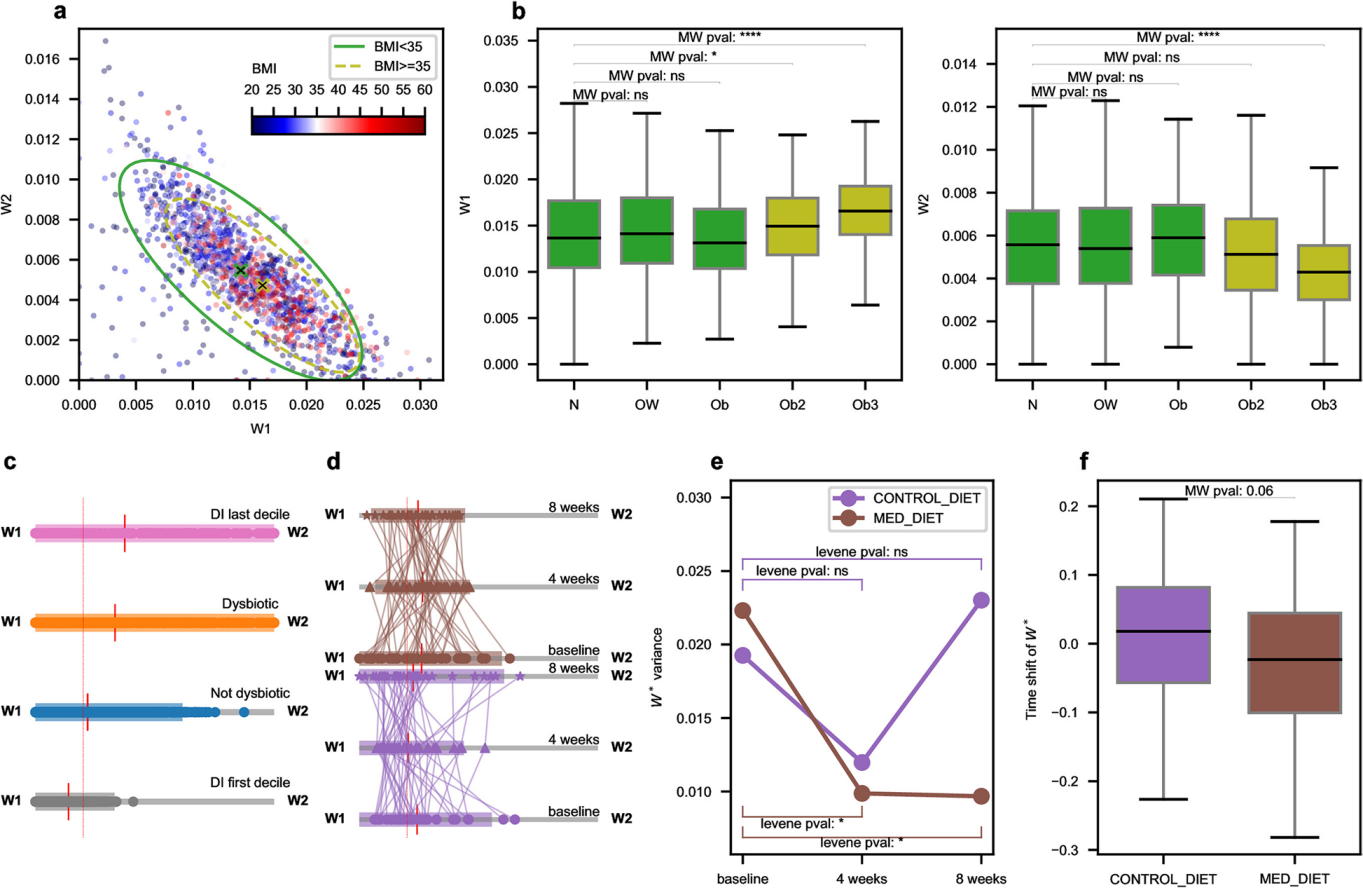
W1 and W2 profile variations. **a** BMI. When BMI is available, samples are displayed in the W1-W2 space, colored by BMI. 95% confidence ellipse are indicated for BMI lower and higher than 35 (class 2, severe obesity threshold). **b** Obesity status. Boxplot of W1 and W2 levels structured by obesity status. We can observe that for highest obesity classes, W1 is significantly higher whereas W2 is significantly lower (MW = Mann-Whitney test). This shift can be also observed in the confidence ellipse centroid in subfigure **a**. **c** Dysbiosis index. All samples are displayed in barycentric coordinates in the W1-W2 space. Barycentric coordinates are equivalent to compute $W^*=W^{(AFT)}_{2}\left/\left(W^{(AFT)}_{1}+W^{(AFT)}_{2}\right)\right.$. The extremity $W^{(AFT)}_{1}$ corresponds to $W^*=0$, i.e. when only profile 1 is present in the sample, and the extremity $W^{(AFT)}_{2}$ corresponds to $W^*=1$, i.e. when only profile 2 is present. Samples are stratified by DI: the first DI decile (gray), non dysbiotic samples (blue, DI<dysbiosis threshold), dysbiotic samples (orange, DI>dysbiosis threshold) and last DI decile (pink) are displayed. The red ticks indicate the group mean, and confidence interval (mean +/- 2*standard deviation) is displayed with a colored bar. The dotted red line indicate the value $W^*=0.2$. We note a higher $W_1-W_2$ unbalance for increasingly dysbiotic groups. **d** Mediterranean diet. Samples are displayed in barycentric coordinates in the $W_1-W_2$ space for the Mediterranean Diet cohort at baseline (circles) , 4 weeks (trianges) and 8 weeks (stars) after intervention for control (mauve) and Mediterranean diet (brown). The mean of each category is displayed with a red vertical line and confidence intervals are indicated as in plot **c**. The dotted red line indicate the value $W^*=0.2$. We can observe that sample variability around the mean is strongly shrunk for the Mediterranean diet group after 4 weeks. **e** Mediterranean diet stabilises the microbiota. The variance of $W_2/(W_1+W_2)$ in the control and Mediterranean diet groups is displayed at baseline, 4 weeks and 8 weeks. The variance decreases for Mediterranean diet after 4 and 8 weeks is significant (Levene test). **e** Time shifts of $W^*$. Time shifts, defined as the difference of $W^*= W_2/(W_1+W_2)$ between 4 weeks and 8 weeks, are displayed with boxplots, for the Mediterranean and control diet groups. Time shifts are reduced after intervention for the Mediterranean group, with low significance ($p = 0.06$, Mann-Whitney one-sided test)

Profiles 1 and 2 particularly contribute to GH production and sugar metabolism AFTs; we therefore wondered if body mass index (BMI) structured the samples in the $W_1$-$W_2$ space (Fig. 5a). When $W^{(AFT)}_{1}$ is high and $W^{(AFT)}_{2}$ is low, higher BMIs are preponderant (light green dashed confidence ellipse), whereas lower BMIs are over-represented in the region defined by low $W^{(AFT)}_{1}$ and high $W^{(AFT)}_{2}$ (green confidence ellipse). Plotting $W^{(AFT)}_{1}$ and $W^{(AFT)}_{2}$ distributions stratified by obesity levels (Fig. 5b) shows that $W^{(AFT)}_{1}$ values are significantly higher and $W^{(AFT)}_{2}$ significantly lower for class 3 obesity compared to healthy samples. Interestingly, the shifts are significantly reversed under statin treatment (Fig. S4f), a drug used against hypercholesterolemia, suggesting metabolism-driven modifications of the microbiota. Statin is known to impact the microbial composition, reducing the prevalence of Bact2 enterotype in patients under treatment [33], consistently with the statin-induced reduction of $W^{(AFT)}_{1}$ since profile 1 is over-represented in Bact2 enterotype (Fig. S4 d). It should be noted that a taxonomic-only profiling of the metagenomes shows a completely different pattern, with a vanishing preponderant taxonomic-only Profile $W^{(AFT)}_{1}$ for higher classes of obesity but small variations of $W^{(AFT)}_{2}$ (Fig. S9e).

As profiles 1 and 2 are preponderant in the samples, we investigated if their respective weights are impacted during dysbiosis. To quantify the balance between profiles 1 and 2 in the microbiota, we introduce the barycentric coordinate $W^*=W^{(AFT)}_{2}\left/\left(W^{(AFT)}_{1}+W^{(AFT)}_{2}\right)\right.$ that we plot with stratification by dysbiosis index (DI, see the “Statistical treatment” section for DI definition). For balanced microbiota (Fig. 5c, blue, DI < dysbiotic threshold), the barycentric coordinates are tightened around an average ratio of 0.2, meaning that profiles 1 and 2 are mixed with a respective ratio 4:1 in non dysbiotic samples. On the contrary, $W^*$ is significantly higher in dysbiotic samples (DI>dysbiotic threshold, orange, $p < 1e-5$, two-sided Mann-Whitney (MW) test), with significantly increased dispersion around the mean ($p < 1e-5$, Levene test). Shrinkage around $W^*=0.2$ is enhanced for the first DI decile (gray) and $W^*$ is more dispersed in the last decile (pink) compared to the set of dysbiotic samples. All together, these observations suggest that dysbiosis is characterized by unbalanced profiles 1 and 2. Furthermore, unbalance is induced by both a significant depletion of profile 1 (Fig. S4 b, MW test) and a significant increase of profile 2 (MW test).

Profile 1 main characteristic is its preponderant contribution in GH-related AFTs, involved in fibre cleavage. We then hypothesized that high fibre diet may impact profile 1 and 2 balance. In an interventional study comparing Mediterranean diet (considered as a high fibre diet) to a control diet, the distribution of the barycentric coordinate $W^*$ are similar in the Mediterranean diet and control groups at baseline (Fig. 5 d). Four weeks after intervention, $W^*$ is tightened around the value 0.2 in the Mediterranean diet group, and this shrinkage is maintained 8 weeks after intervention, whereas the dispersion is similar to the baseline in the control group (Fig. 5d). Furthermore, the variance of $W^*$ is significantly reduced after intervention in the Mediterranean diet (Fig. 5e, Levene test) unlike the control group. The shift of $W^*$ between four and eight weeks are higher in the control group compared to the Mediterranean diet (Fig. 5d) with slight significance ($p = 0.06$, one-sided MW test). These observations suggest that the higher fibre intake in the Mediterranean diet contributes to the stabilization of profile 1 and 2, particularly equipped with fibre degradation functions, around a non-dysbiotic ratio.

### Profiles 3 is associated to Crohn’s disease and profile 4 to slow transit

**Fig. 6:**
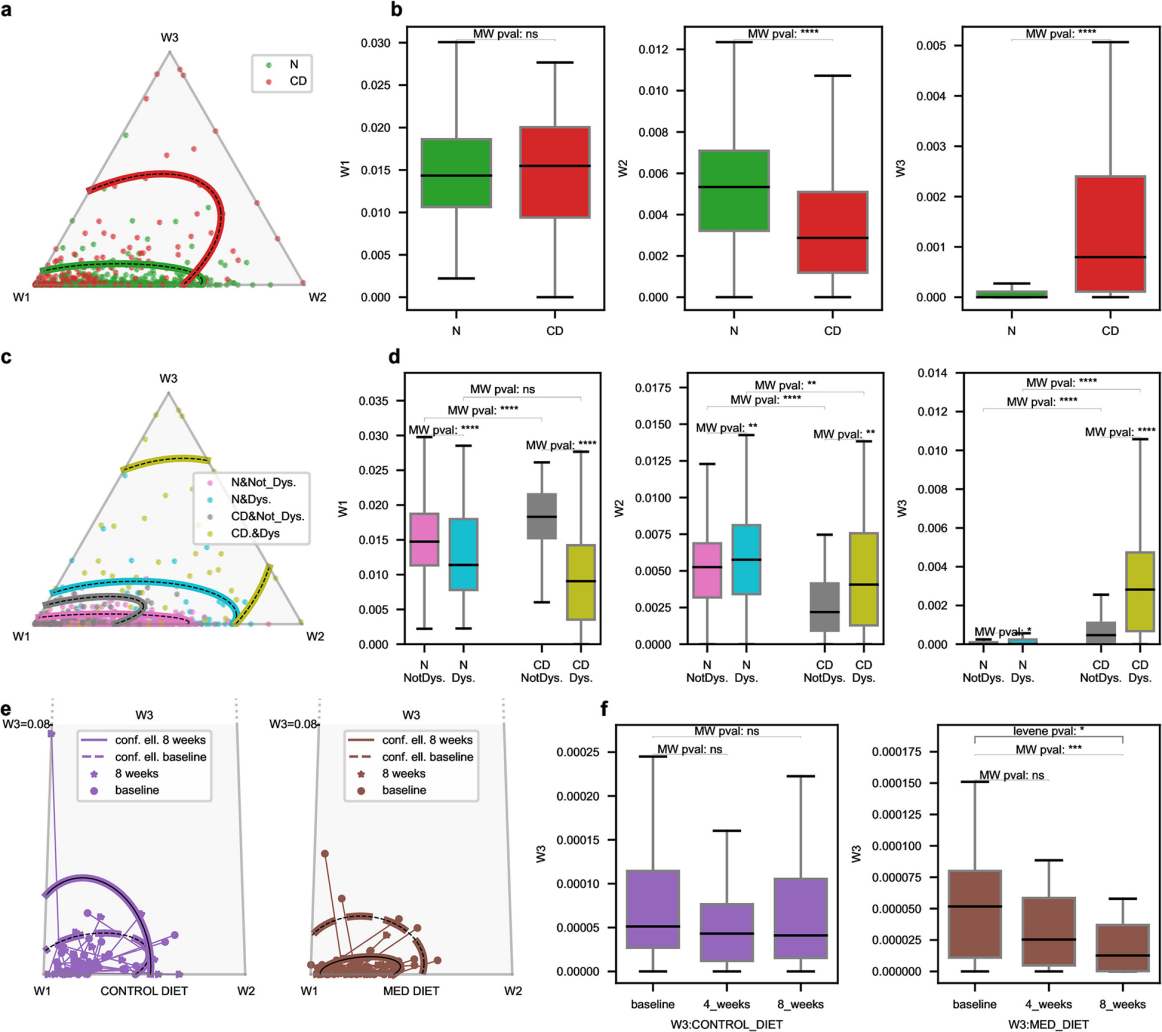
$W_3$ Profile variations associated to inflammatory status. **a** Crohn’s disease. Ternary plot in the $W_1-W_2-W_3$ space of samples colored by disease status (red: Crohn’s disease (CD), green: Non-CD). 95% confidence area of each category are displayed with plain lines. **b** Boxplot of $W_1$, $W_2$ and $W_3$ levels, structured by disease status. We can observe that CD samples have no marked difference in $W_1$ levels but are characterized by significantly lower $W_2$ and strongly higher $W_3$ levels. This pattern differs from dysbiotic samples where $W_2$ were over-represented. This observation corroborates the shift of the confidence area in the ternary plot **c**. **c** Unraveling dysbiotic and CD profiles. CD and healthy (N) dysbiotic (dys.) and not-dysbiotic(Not_Dys.) samples are displayed in a ternary plot in the $W_1-W_2-W_3$ space. **d** Boxplot of the $W_1$, $W_2$ and $W_3$ levels, structured by dysbiotic and CD status. **e** Mediterranean diet. Ternary plots in the $W_1-W_2-W_3$ space of samples of the Mediterranean diet cohort. Control and Mediterranean diet groups are displayed in separated ternary plots. For a same individual, samples at baseline (circles) and 8 weeks after intervention (stars) are represented and linked by a line, showing the individual time trajectory. 95% confidence areas are displayed for baseline and 8 weeks groups. Ternary plots are clipped in the $W_3$ direction at W3 = 0.08. **f** Boxplots of $W_3$ levels in the control and Mediterranean diet groups at baseline, 4 weeks and 8 weeks after intervention. $W_3$ mean and variance are significantly reduced after 8 weeks of Mediterranean diet

When plotting the weight of the 3 first profiles in a ternary plot in the $W_1-W_2-W_3$ space (Fig. 6 a), Crohn’s disease (CD) samples (red dots, red line : 95% confidence) are mainly shifted towards the $W_1$ and the $W_3$ corners, whereas healthy samples (green dots, green line: 95% confidence) are kept near the basis of the triangle, around the ratio 0.2 between profiles 1 and 2 previously identified as a marker of healthy samples. This means that Crohn’s disease is characterized by unbalanced profiles 1 and 2 and over represented profile 3. Bar plots (Fig. 6b) shows that the unbalance is driven by a very significant (MW test) depletion of $W^{(AFT)}_{2}$ in CD samples while $W^{(AFT)}_{1}$ is not significantly modified and a very significant increase of $W^{(AFT)}_{3}$ is observed (MW test). Hence, in CD samples, a shift in the profiles weights occurs from profile 2 towards profile 3. This shift carries enough signal to correctly classify CD and healthy samples using SVM classifier with high accuracy (Fig. S5 e., recall: 0.94, precision: 0.81, AUC: 0.92 for the unseen test cohort).

This observation is unexpected since dysbiotic samples ought to be over-represented in CD samples and we just saw that dysbiosis is characterized by an increase of $W^{(AFT)}_{2}$ (Fig. S4 a and b). We then color-coded dysbiotic and not dysbiotic samples in the ternary plot (Fig. 6c) and stratified accordingly the bar plots (Fig. 6d). In not dysbiotic samples, the weight of profiles 1 is increased in CD compared to healthy population whereas profile 2 drops, with high significance. During dysbiosis, usual shifts occur: $W^{(AFT)}_{1}$ is reduced while $W^{(AFT)}_{2}$ is increased in both CD and normal populations, but $W^{(AFT)}_{2}$ remains lower for dysbiotic CD compared to healthy dysbiotic samples, with high significance (MW test). We also observe that profile 3 is not a strong marker of dysbiosis since in healthy populations, a dysbiosis only triggers a limited increase of profile 3 weight, while CD induces a strong increase of $W^{(AFT)}_{3}$ whatever the dysbiotic status with a strong enhancement during dysbiosis (Fig. 6 d). Interestingly, profile 3 is mitigated by Mediterranean diet (Fig. 6f). After 8 weeks of high-fibre diet, profile 3 is significantly reduced (MW test) together with its variance (Levene test) compared to baseline and to control (MW test $p=3e-3$, Levene test $p=4e-2$) so that samples are kept near the basis of a ternary plot (Fig. 6 e). Mediterranean diet has been shown to improve the inflammatory status of patients experiencing an increase of microbial richness after diet change[34], suggesting that $W^{(AFT)}_{3}$ reduction after intervention could be linked with the inflammation reduction. This would be consistent with the taxonomic composition of profile 3, carrying *Proteobacteria* known to bloom during inflammation [80]. The association of $W^{(AFT)}_{3}$ with the CD inflammatory disease and the over-representation of higher $W^{(AFT)}_{3}$ in Bact2 enterotype (Fig. S4d) are also consistent with the previous identification of Bact2 as a dysbiotic microbiome [33].

**Fig. 7:**
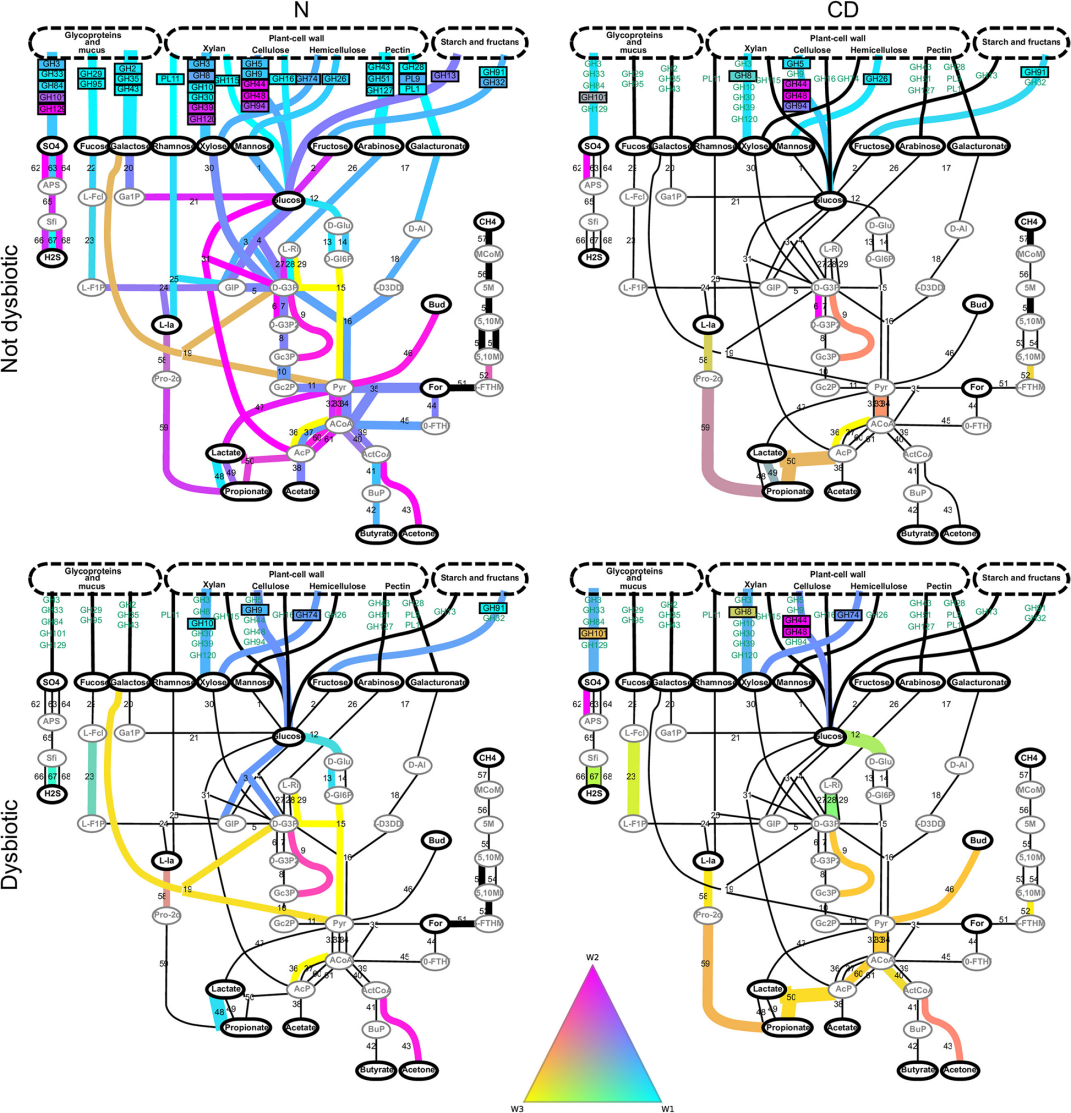
Variation of profiles contributions in healthy vs CD and dysbiotic vs not dysbiotic samples. The metabolic network of fibre degradation is displayed, and profile contribution in GH/PL and KO counts is color coded on the corresponding arrows of the network. Profile contributions are displayed for healthy (N, left panel) and CD (right panel) samples and dysbiotic (lower panel) and not dysbiotic (upper panel) samples. Namely, we compute CD and healthy average profiles weight $\bar{W}^{(AFT)}_{train,g}$ by averaging $W^{(AFT)}_{train}$ on the sample group *g* (N and dysbiotic, N and not dysbiotic, CD and dysbiotic, CD and not dysbiotic). Average AFT counts $\bar{X}^{(AFT)}_{train,g}$ are obtained in the same manner for each group. Then, average profile contribution for AFT *j* and profile *i* is computed with $\bar{W}^{(AFT)}_{train,g,i} H^{(AFT)}_{ij}\left/\bar{X}^{(AFT)}_{train,g,j}\right.$. The respective relative contribution of profiles 1, 2 and 3 is then mapped into a ternary color map (central triangle) and displayed on the corresponding arrow or GH/PL box. Black arrows indicate AFT the main contribution of which is given by profile 4. Arrow widths are proportional to AFT counts in $\bar{X}^{(AFT)}_{train,g}$. For N&Not dysbiotic graph, all the AFTs are represented (control situation). For the other graph, the AFTs that significantly changed compared to N&Not dysbiotic group (*t*-test and Benjamini Hochberg correction with $FDR < 0.05$) were filtered; we then ordered AFTs by compositional changes compared to N&Not dysbiotic group (L2 difference on $\bar{W}^{(AFT)}_{train,g,i} H^{(AFT)}_{ij}\left/\bar{X}^{(AFT)}_{train,g,j}\right.$ computed on both groups) and kept the top 20 AFTs in order to highlight the main changes in microbiota composition. The same figure can be explored dynamically (see Additional file 10—metabolic exploration)

The weight transfer from profile 2 towards profile 3 reflects functional shifts in CD compared to healthy samples. Functional modules are significantly over-represented in CD samples compared to healthy ones, in particular ED, fucose, galactose GH, sulfur and propionate pathways (Fig. S5a and c, fdr 0.05, Benjamini-Hochberg correction). A closer look to the metabolic pathaways during CD and dysbiosis indicates a shift towards non typical metabolic pathways in the metagenome (Fig. 7). If some GHs are shifted, mainly involved in cellulose (GHs 44 and 48), xylan (GH 8) and glycoprotein (GH 101) degradations, the most interesting modifications occur in the KOs. First, the downstream part of fucose pathway including the propane1-2 diol production from L-lactadehyde (AFT 58; K13922) and propionate production through AFT 59 is particulary marked in CD samples: it is a propionate production pathway distinct to the usual one based on lactate transformation, which is reduced in CD (AFT 50). Consistently, the genes involved in acetate production through AFT 36 (K04020) and 60 (K13788) are non typical for anaerobic pathways and are over-represented in CD samples. Further shifts are observed during CD presenting alternatives in sulfur (AFT 62), SP-ED (AFT 9) and pyruvate (AFT 33) pathways.

During dysbiosis, these shifts are further enhanced. Fucose fermentation pathway is exacerbated with the increase of AFT 23 encoding for fucK which is present in *Proteobacteria* and *Akkermansia muciniphila* genomes (Fig. S6), which complete a pathway from fucose to propionate and enforces the availability of the corresponding genes. AFT 67 encoding for sulfite and NAPDH from hydrogen sulfide (Fig. 7) driving hydrogen removal from dissimilatory sulfate reduction is also increased: these functions are characteristic of *Proteobacteria* and *Bacteroidota* (Fig. S6) and are an alternative to AFTs 66 and 68 more present in *Firmicutes* and profile 2 in healthy samples (Fig. 7, S6 and Additional file 10—metabolic exploration). Further modifications occur during dysbiosis and dysbiotic CD such as AFT 43 (acetone production) or GH 74 (hemicellulose degradation). Alternatively, some shifts are preponderant in healthy dysbiotic samples but do not belong to the main modifications in dysbiotic CD. Among them, alpha-galactose fermentation as characterized by AFT 19 (including gene dgoK; 2-dehydro-3-deoxygalactonokinase [EC:2.7.1.58]) involved in galactose to pyruvate metabolism is an alternative to galactose transformation towards glucose during dysbiosis. The alternative ED pathways for glucose fermentation is also enhanced (AFTs 12, 13 and 15) compared to EMP pathway in healthy dysbiotic samples, since these AFTs are over-represented in profile 3 (Fig. 3a and Additional file 10—metabolic exploration).

If the functional count changes are relatively limited (Fig. S5c), the taxonomic changes are massive (Fig. S5d), supporting the fact that the observed functional shifts are carried by modifications in the taxonomic composition of the microbiota during CD. Functional redundancies across micro-organisms (Fig. S6) lead to limited changes in the functional composition of the fibre-related metagenome, with more marked modifications in a limited number of functions involved in species functional specialization in alternative pathways. For example, *Proteobacteria* are characterized by the presence of propionate-related AFTs (Fig. S6), which relates the preponderance of *Proteobacteria* in CD samples (Fig. S5d) to the shift towards $W^{(AFT)}_{3}$ in the distribution of propionate-related AFTs during CD (AFT 58, 59, 50, Fig. 7). However, taxonomic-only profiling of the metagenome does not allow to accurately reproduce the stratification between dysbiotic and CD samples obtained with Profile 2 and Profile 3 (Fig. 6d and Fig. S9g).

Regarding profile 4, $W^{(AFT)}_{4}$ weight is significantly reduced for higher Bristol scores (3 to 7), associated to more fluid stools, compared to low Bristol scores (1–2) associated to hard stools (Fig. S7a). As fluid stools are often related to lower retention times in the gut, we wondered if larger retention times would favour profile 4 and investigated a cohort including patients suffering Parkinson’s disease, a disease associated to constipation, reported in 80–90% of PD patients [81]. As expected, $W^{(AFT)}_{4}$ is higher in PD samples compared to control with slight significance (MW test, *p*: 5.3e−2, Fig. S7,b). This relation of $W^{(AFT)}_{4}$ with low transit time can be linked to the taxonomic composition of profile 4, mainly marked by the presence of methanogenic archaea, characterized by low growth rates.

## Discussion

We used the NMF method previously introduced [25] to analyse metagenomic gene count matrix taking into account prior knowledge on fibre degradation. Our approach is based on a two-step microbiota simplification. In the first step, functional marker genes of interest are selected to build the AFT count matrix while providing a simplified view of the metagenome focused on fibre degradation. In the second step co-varying AFT are identified using NMF, leading to 4 universal functional profiles that can be used to reconstruct external samples. This double simplification is crucial to decipher changes among the very high dimensional metagenomic data and to provide extensive biological interpretations of the different profiles and their shifts during diseases. This functional viewpoint is supplemented by a taxonomic make up of the 4 profiles. Several external datasets were further studied and profiles weights variations were linked to obesity, dysbiosis, Mediterranean diet, statin intake and Crohn’s disease. The expression of these universal functional profiles has been checked in metatranscriptomic data, and their taxonomic characterizations have been compared to a taxonomic-only NMF profiling of the metagenomes.

Screening the profiles weights allows the identification of global shifts in the microbiota induced by conjoint changes in the co-varying genes of the profiles. We emphasize that this differential analysis relies on four quantities only (the weights of the four profiles), representing a dramatic reduction of the dimensionality. Furthermore, as the profiles take in charge specific parts of the metabolic network of fibre degradation, our framework is very suitable for functional interpretation: the profiles weights variations are directly linked to functional variations that can be mapped to specific metabolic pathways of the fibre degradation network. Finally, the profiles functional potentials are particularly consistent with their taxonomic composition and the functional peculiarities of the genomes they include.

In particular, new biomarkers were identified for dysbiosis and CD. A healthy microbiota is characterized by a balance of profiles 1 and 2 around a proportion 4:1 while microbiota diverging from this 4:1 proportion are over-represented in dysbiotic samples. As profiles 1 and 2 mainly differ by their GHs, these shifts reflect preponderantly changes in fibre cleavage. In the same way, profile 2 and profile 3 are sufficient to classify CD samples with high accuracy and reflect functional shift from usual to unusual pathways for fucose, propionate, H2S, SPED, acetone or butanediol, together with a bloom of *Proteobacteria*. These biomarkers give in themselves new insights on the underlying ecology during these pathological events. However, due to our focus on fibre degradation, we only capture changes inside fibre cleavage and fermentation pathways of fibre-derived sugars: our methodology is missing all the functional shifts outside this scope, which can be important in particular in pathological situations. This limitation could explain why many samples are tagged as dysbiotic with the 9.9M genes pBCd-derived classification but display a healthy ratio of 4:1 between profiles 1 and 2 for fibre-related genes. Hence, our methodology could be extended to other metabolic functions, such as respiration functions in micro-aerophilic environments during inflammation or glycoprotein degradation, or to non-metabolic functions such as antibiotic production or bile salt hydrolysis.

AFT selection is a crucial step of this methodology. Narrowing down the number of genes in the metagenome is needed for microbiota simplification. Furthermore, the careful selection of specific genes allows to link an AFT count to specific metabolic pathways despite ubiquitous genes: enlarging too much the set of selected genes would have blurred the biological interpretation by adding genes involved in very different pathways. However, some of these genes had to be added in the selection to allow certain degradation pathways. Selection step is then a trade-off between specificity and completeness of the global network, in the context of ubiquitous enzymes. This is particularly true for the first step of the degradation pathway, from dietary and host-derived fibres to simple sugars, due to the large diversity of fibres [82], and the presence of GHs with a broad range of cleaving sites [37, 38]. Again, this modelling option can be seen as a bias of the present study that could be corrected by enlarging the functional scope of the method by enrolling other functions, e.g. to put the focus on the metabolism by the microbiota of glycosaminoglycans (GAGs), which are important for the colonization of certain gut bacteria but also for the host health, GH88, PL29, PL8 and PL33 should be added to the upper part of the metabolic network, with connections to galactose [83]. We consider this trade-off as a necessary bias intrinsic to microbiota simplification, a price to pay for facilitated biological interpretations.

Another ambition of microbiota simplification is to decipher universal pattern, or functional invariant that can be searched for in a metagenomic sample. In the present study, four functional profiles are identified that structure the main part of the metagenomic samples. In the inference procedure, strong caution has been put in hyperparameter selection and inference validation, with a particular criterion on the stability of the inferred matrix H: the selected hyperparameters reduced the sensitivity of the inferred H to subsampling of the training set, enforcing the universality of the inferred profiles. Furthermore, the training set has been carefully constituted by enrolling a large panel of healthy, inflammatory disease, metabolic disorder, with a strong caution not to introduce age, sex, study or origin bias. The representativeness of the training set has been validated a posteriori by checking that its intrinsic pBCd distribution was identical to the overall pBCd distribution. We also stress that the ability of the profiles to accurately reconstruct external samples has been widely validated by applying them to 2571 unseen samples from 5 external studies. However, other inference settings such as other regularization penalty, or a different learning set, could bring slightly different profiles. This drawback is inherent to dimension reduction strategies, also present in other strategies such as enterotyping: the microbiota simplification allows to decipher general features but the statistical method itself comes with intrinsic bias that introduces peculiarities.

A classical debate in microbial ecology is the cross-talk between taxonomic composition of the microtiota and its functions. As functions are derived from the taxa present in the sample, most of the microbiota simplification efforts have been put on exhibiting patterns of diversity, i.e. invariants in the taxonomic composition of the samples. This led to the concept of enterotypes [20, 84] and more recently of enterosignatures [85]. Here, we proposed to reverse the view point by first exhibiting functional profiles and then digging into taxonomic entities that could carry the functions they involve. From a microbial ecology perspective, this approach is similar as identifying stable environment-dependent niches, i.e. the metabolites and their related functions in a Profile, that can be occupied by adapted microbes, i.e. the taxonomic make-up of the profiles. In this study, we compared both microbiota decomposition approaches. Function-based reduction seems to be more general in the sense that functional profiles prevalence is rather stable among datasets (Fig. S1j) compared to taxonomic-only reduction (Fig. S9c) that shows exclusion of taxonomic profiles in some datasets (e.g. $W^{(PG,nmf)}_{2}$ and $W^{(PG,nmf)}_{4}$ in hmp2 dataset). This feature is expected since the gut environment is strongly constrained by the host physiology and the diet, whereas taxonomic composition is also subject to contingency, such as temporal priority effect [86, 87] or biogeography effects [88, 89]. Taxonomic-only partitioning such as enterotypes proved to be efficient in structuring populations with respect to geographic origin [90], age [85], diet [91, 92] or disease [33, 90, 93, 94]. However, the present study shows that the function-based microbiota simplification allows capturing weak signals such as stratifying between dysbiotic or undysbiotic CD samples (Fig. 6c, d) or deciphering a functional switch in higher classes of obesity (Fig. 5b), contrary to taxonomic-only profiles (respectively Fig. S9f, g and e). But the main advantage of this function-based approach is the functional interpretation of the shifts in the metagenomes induced by profiles weights variations. They facilitate the identification of key functions that can be targeted to manipulate the microbiota with pre- or probiotic treatments.

The NMF method was previously used for metagenomic data analysis [95–97]. It can also be related to other dimension reduction or soft clustering techniques. NMF is comparable from a modelling point of view to mixture models such as DDM, that were used to identify enterotypes [20]: the metagenomic counts are seen as a mixture of different populations the composition and weight of which is unknown. The inference setting is however very different, and NMF suggests a continuous interpretation of the weights, by comparison to discrete allocation to an enterotype in DMM. NMF method can also be interpreted as a PCA-like method, constrained by the positiveness of the weights and the direction. The very specific added-value of our approach compared to previous microbiota reduction methods is the inclusion of prior knowledge on microbial physiology and bio-chemistry in the inference process through the functional constraint *F* (see Eq.(1)). This introduction, deeply discussed in [25], facilitates the biological interpretation of the profiles, compared to completely agnostic approaches. We believe that adding such modelling overlay on statistical learning methods could be decisive in facilitating the integration of the wealth of knowledge acquired during decades by microbiologists before the omics revolution in the analysis of the high-throughput data of NGS methods.

## Conclusion

In this paper, we analysed a large amount of data coming from various mNGS studies. From a training dataset with 1153 samples from 7 cohorts, we performed a two step microbiota simplification method based on AFT selection and NMF dimension reduction technique. We identified four universal functional profiles that were thoroughly validated on 2571 external samples from 5 independent studies and further characterized in term of functional capabilities related to fibre degradation and taxonomic composition. Profile 1 is strongly equipped in GH, making hydrolysis of a large variety of carbohydrates its main characteristic, and is mainly composed of *Bacteroidetes*. By contrast, profile 2 is more directed towards starch or glycoprotein degradation and is mainly composed of *Firmicutes*. Profile 1 and 2 balance of roughly 4:1 is associated with a healthy microbiota, while unbalance is associated with dysbiotic events. A Mediterranean diet can help stabilizing the microbiota around this healthy equilibrium. Profile 1 and 2 unbalances mainly reflect shifts in fibre cleavage towards simple sugars, with GH distribution being the principal difference between these profiles.

Profile 3 takes over profile 2 during CD, making shifts between both profiles a biomarker able to correctly classify CD patients. This ecological unbalance reflects functional reorientations towards unusual metabolism, in particular for fucose and H2S degradation and propionate, acetone and butanediol production. These alternative pathways are carried by *Proteobacteria*, the main phylum involved in profile 3. Profile 4 is mainly marked by rare metabolism, such as methanogenesis, and is favoured by slow transit.

Integrating anaerobic microbiology knowledge into statistical learning methods narrows down the metagenomic analysis to investigating ecosystem traits and identifying functional invariants that can be easily monitored to identify markers of diet, dysbiosis, inflammation and disease.

### Supplementary Information

**Additional file 1:** **Figure S1.** AFT reconstruction error distribution and weight distribution. The relative reconstruction error distribution among samples defined as $\left\| X^{(AFT)}_{g,i} - W^{(AFT)}_{g,i}H^{(AFT)}\right\| \left/\left\| X^{(AFT)}_{g,i}\right\|\right.$ is displayed, and structured according to the different groups *g* encountered along the study i.e. a) datasets, b) obesity status, c) enterotypes, d) dysbiotic status, e) statin intake, f) Bristol score, g) Crohn disease status, h) Mediterranean or control diet and i) parkinson disease. For comparison, the distribution observed in the train dataset is displayed in all graphs (gray dash lines), together with its mean relative reconstruction error (red dashed line). The mean and quantile 90% of each distribution are displayed with the vertical red and black lines. We can see that the relative reconstruction error distributions are very homogenenous along every structuring variables, except for dysbiotic and CD samples and Prevotela enterotypes, where relative reconstruction error is increased, but keeping the 95% quantile under 44% of reconstruction error. All together, the functional profiles allow to reconstruct the large majority of external samples with a level of accuracy comparable to the training dataset reconstruction, with a higher bias for dysbiotic, CD and Prevotela samples. j) The distribution of the weights $W^{(AFT)}_{i}$ are displayed for each dataset, with violin plots in log scale. profiles 1 and 2 are preponderant, and Profile 3 and 4 are associated with lower weights.

**Additional file 2:** **Figure S2.** Top functional and taxonomic profiles contribution to metagenome. The top 50 relative profiles contribution to a) AFTs b) PGs and c) MGS-derived genus reconstruction are displayed. Namely, we compute for Profile *i* and AFT or genome *j* the profiles contribution $\bar{W}^{(AFT)}_{train,i}H^{(AFT)}_j \left/\bar{X}^{(AFT)}_{train,j}\right.$ where $\bar{W}^{(AFT)}_{train}$ and $\bar{X}^{(AFT)}_{train}$ are averaged among the training samples. Then, contributions are sorted and top 50 contributions are kept and colorcoded by KO or GH for AFT, and phylum for PGs and MGS clustered by genus. Profile 1 is characterized by an over-representation of GH and *Bacteroidetes*, while Profile 2 is characterized by more KOs, and *Firmicutes* and *Actinobacteriota*.

**Additional file 3:** **Figure S3.** Phyla reconstruction error distribution when reconstructing the PG counts. The phyla relative reconstruction error distribution among samples defined as $\left\| \left(X^{(PG)}_{i} - W^{(AFT)}_{i}H^{(PG)}\right).A_{phyla}\right\| \left/\left\| X^{(PG)}_{i}.A_{phyla}\right\|\right.$ is displayed, where $X^{(PG)}$ is the count matrix of the 203 representative genomes and $A_{phyla}$ is an allocation matrix of each genome to its phyla, and structured according to the different classes encountered along the study, i.e. a) datasets, b) obesity status, c) enterotypes, d) dysbiotic status, e) statin intake, f) Bristol score, g) Chron disease status, h) Mediterranean or control diet and i) parkinson disease. For comparison, the distribution observed in the train dataset is displayed in all graphs (gray dash lines), together with its mean relative reconstruction error (red dashed line). The mean and quantile 90% of each distribution are displayed with the vertical red and black lines. We can see that the relative reconstruction error distributions of the phyla are very homogenenous along every structuring variables, except for dysbiotic, CD and Parkinson disease samples, where relative reconstruction error is increased. Like for AFTs, the functional profiles allow to reconstruct the taxonomic composition of the large majority of external samples at the phyla level with a level of accuracy comparable to the training dataset reconstruction. j) MGS. The same procedure is repeated with MGS. Namely, $\left\| \left(X^{(mgs)}_{i} - W^{(AFT)}_{i}H^{(mgs)}\right).A_{phyla}\right\| \left/\left\| X^{(mgs)}_{i}.A_{phyla}\right\|\right.$ is displayed, where $X^{(mgs)}$ is the MGS count matrix and $A_{phyla}$ is an allocation matrix of each MGS to its phyla and structured according to the different classes encountered in the ’train’ test, i.e. k) dysbiotic status and l) Chron disease status. The MGS count matrix are correctly reconstructed, whatever the structuring variable.

**Additional file 4:** **Figure S4.** Characterization of dysbiosis, enterotypes and statin related samples. a) Dysbiosis. Ternary plot in the $W_1-W_2-W_3$ space of samples colored by dysbiotic status. We also display the 95% confidence area for each category (colored line). b) Boxplot of $W^{(AFT)}_{1}$, $W^{(AFT)}_{2}$, $W^{(AFT)}_{3}$ and $W_4^{(AFT)}$  ﻿levels, structured by dysbiotic status. We can observe that dysbiotic samples are characterized by significantly lower $W^{(AFT)}_{1}$, higher $W^{(AFT)}_{2}$ and strongly higher $W^{(AFT)}_{3}$ levels. This information corroborates the much wider confidence area for dysbiotic samples in the ternary plot a). c) Enterotypes. Samples are displayed in a ternary plot in the $W_1-W_2-W_3$ space, colored by enterotype, when available. 95% confidence ellipses of each class are displayed. d) Boxplot of $W^{(AFT)}_{1}$, $W^{(AFT)}_{2}$, $W^{(AFT)}_{3}$ and $W_4^{(AFT)}$  levels, structured by enterotypes. We can observe that Ruminococcus enterotype is overrepresented for higher $W^{(AFT)}_{2}$ and lower $W^{(AFT)}_{1}$. The reverse observation can be made for Bact2 enterotype. To a lower extent, Bact1 enterotype is more prevalent for lower $W^{(AFT)}_{1}$ and higher $W^{(AFT)}_{2}$, which is the inverse of Prevotella enterotype. High $W^{(AFT)}_{3}$ counts are related to Bact2 enterotype. e) Statin. Ternary plot in the $W_1-W_2-W_3$ space, colored by statin intake, together with 95% confidence ellipses. f) Boxplot of $W^{(AFT)}_{1}$, $W^{(AFT)}_{2}$ and $W^{(AFT)}_{3}$ levels structured by statin intake. $W^{(AFT)}_{1}$ is significantly lower for statin intake, whereas $W^{(AFT)}_{2}$ is significantly higher. No significant shift is observed for $W^{(AFT)}_{3}$.

**Additional file 5:** **Figure S5.** CD-related profiles characterization. a) Functional differential analysis between CD and healthy samples (N). Average profile contribution in the significantly different functional module frequencies for CD and N groups. Functional modules are defined in fig. 1. a. We averaged the $L_1$ normalized $W^{(AFT)}$ (resp. $X^{(AFT)}$) for the CD and N groups of the train dataset, noted $\bar{W}^{(AFT)}_{train,L_1,g}$ (resp. $\bar{X}^{(AFT)}_{train,L_1,g}$) for $g=CD$ or *N*, and computed $\bar{W}^{(AFT)}_{train,L_1,g} H^{(AFT)}$. We then gathered the columns of $\bar{X}^{(AFT)}_{train,L_1,g}$ by functional modules and filtered functions with significant changes (t-test, 0.05 fdr Benjamini-hochberg correction) between N and CD groups. For selected modules, we computed $\left.\bar{W}^{(AFT)}_{train,L_1,g,I}H_{Ij}\right/\sum _i \bar{W}^{(AFT)}_{train,L_1,g,i}H_{ij}$, for Profile *I*, group $g=CD$ or *N*, and functional module *j*, displayed in barplots, in order to display profile contribution for each functional module. b) Taxonomic differential analysis between CD and healthy samples. The same procedure is repeated on phyla counts. After computing $\bar{X}^{(PG)}_{train,L_1,g}$ and pooled representative genome counts by phyla, the significantly varying phyla (t-test, 0.05 fdr Benjamini-hochberg correction) between N and CD groups are filtered. Then, $\left.\bar{W}^{(PG)}_{train,L_1,g,I}H^{(PG)}_{Ij}\right/\sum _i \bar{W}^{(PG)}_{train,L_1,g,i}HPG_{ij}$, for Profile *I*, group $g=CD$ or *N*, and functional module *j*, is displayed in barplots, in order to display the Profile contribution to the reconstruction of each phyla. c) Log2 ratio between CD and N groups of filtered functional groups are displayed. d) Log2 ratio between CD and N groups of filtered phyla are displayed. Whereas functional variations are limited, taxonomic variations are more acute. e) Classification of CD samples. The SVM classifier for CD/N, trained on the hmp2 cohort, is displayed in the $W_2-W_3$ space (normalised with min-max scaling). The black line separates the negative (green) and the positive region (red). The samples of the ‘hmp2’ (train, crosses) and ’CD’ (test, circles) cohorts are displayed, colored by disease status. We observe that $W_2-W_3$ variations for CD samples are strong enough to capture this signal with a classifier (recall: 0.94, precision: 0.81, AUC: 0.92 for the CD unseen cohort).

**Additional file 6:** **Figure S6.** Prevalent genomes functional profiling. Selected AFTs are annotated in the prevalent genomes and presence/absence is displayed (middle panel), sorted by functional blocks (top). The genome names are indicated (short name and NCBI ID, right panel), colorcoded by phylum, and the the taxonomic allocation of the genomes in profiles is indicated by the presence/absence matrix in $H^{(PG)}$ (right panel, taxo. alloc. Profile *i* is the *i*-th column of this matrix). The 4 profiles are added to the genome list and displayed with presence/absence tags (a KO is assumed present in the Profile if its frequence is higher than $1e-3$). Hierarchical clustering is performed ($k=4$ clusters), based on pairwise-Jaccard distance computed on AFT presence/absence matrix (corresponding dendogram in the left panel), and genomes are sorted accordingly in the middle and right panels. We note that the 4 profiles are clustered at the same time than the genomes. *Bacteroidetes* and *Actinobacteria* are gathered into their own cluster (orange and green clusters), whereas *Firmicutes* are splitted in two clusters: the main part is clustered with *Proteobacteria* (red), while the others are clustered with less prevalent phyla such as *Desulfobacterota* or *Euryarchaeota* (purple), the separation being based on difference in presence of ED and SPED-related AFTs. Profile 1 clusters with *Bacteroidetes*, consistantly with its taxonomic profiling (Fig. 4). This cluster is marked by higher presence of GH and PL, consistantly with its functional profiling (Fig. 3). Profile 2 and 3 cluster with *Firmicutes* (red cluster), profile 3 being included in a sub-cluster involving *Proteobacteria*, again consistantly with their respective taxonomic profiling (Fig. 4). Profile 4 clusters with methanogens (*Euryarchaeota*) as expected. This last cluster is characterized by lower presence of GH/PL in the genomes.

**Additional file 7:** **Figure S7.** Profile 4 association with Bristol score and Parkinson’s disease. a) Bristol score. Boxplot of $W^{(AFT)}_{4}$ levels, structured by Bristol stool score. b) Parkinson’s disease. Boxplot of $W^{(AFT)}_{4}$ levels in PD and healthy control samples. We can observe that the significance of the difference between groups is slight ($p=5.3e-2$, MW test) c) Ternary plot in the $W_1-W_2-W_4$ space, colored by Bristol stool score. 95% confidence ellipses of each class are displayed. d) Ternary plot in the $W_1-W_2-W_4$ space of PD and healthy control samples. 95% confidence ellipses of each class are displayed.

**﻿Additional file 8:** **Figure S8.** Expression profiling by searching for transcripts co-varying with AFT profiles in metatranscriptomics data. a) Reconstruction error distribution. Metatranscriptomics data are acquired from the hmp2 database, and an AFT expression count matrix $X^{(AFT,mtx)}$ is assembled. Expression profiles are constructed by computing *Hmtx* such that $X^{(AFT,mtx)} \simeq W^{(AFT)}_{H^{(AFT,mtx)}}$ by NNLS, i.e. by searching for AFT expressions that co-varies with the AFT profiles $H^{(AFT)}$. Relative reconstruction error distribution among samples defined as $\left.\left\| X^{(AFT,mtx)}{g,i} - W^{(AFT)}_{g,i}H^{(AFT,mtx)}\right\| \right/\left\| X^{(AFT,mtx)}{g,i}\right\|$ is displayed, and structured according to the different groups *g* encountered in the hmp2 dataset i.e. dysbiotic, non dysbiotic, Chron’s disease and healthy patients. The vertical dotted red line show the average reconstruction error on AFT metagenomic counts in the training set. The vertical red line shows the average reconstruction error on AFT metatranscriptomic data in each group. We can see that the reconstruction is less accurate than in metagenomics, but is still kept in a reasonable level. b) KO and GH-related AFT expression frequencies are first gathered to show the distribution of KO and GH in each Profile (top central pie chart). Then, the frequency of each AFT expression is renormalized by KO or GH/PL total frequency, and displayed in pie-charts for KO (left) and GH/PL (right) after clustering by functional modules (color coded, see Fig. 1a for the functional modules). The number of the KO or GH-related AFT is displayed in its corresponding pie-chart sector (radially, inner zone) when its frequency is higher than 3% in the profile. We can observe that this expression profiles are very similar to the AFT profiles in Fig. 3, specially for the preponderant profiles 1 and 2.

**Additional file 9:** **Figure S9.** Taxonomy-only profiling of the metagenomes. We performed a NMF on the $X^{(PG)}$ and $X^{(mgs)}$ taxonomy count matrices to recover the couples $\left(W^{(mgs,nmf)}_{train},H^{(PG,nmf)}\right)$ and $\left(W^{(mgs,nmf)}_{train},H^{(mgs,nmf)}\right)$ so that $X^{(PG)}_{train} \simeq W^{(PG,nmf)}_{train} H^{(PG,nmf)}$ and $X^{(mgs)}_{train} \simeq W^{(mgs,nmf)}_{train} H^{(mgs,nmf)}$. We then acquired the weights matrices $W^{(PG,nmf)}_{i}$ by NNLS of $X^{(PG)}_{i}$ wrt $H^{(PG,nmf)}$ for $i \in \{hmp2,CD,metacardis,med.diet,Parkinson\}$. a) The 203 genomes frequencies in $H^{(PG,nmf)}$ are displayed in pie-charts and clustered by successive taxonomic levels, i.e. taxa (outer ring), genus, class and phyla (inner ring), color-coded by phyla (phyla name displayed radially in the outermost zone). Taxa names are displayed radially when their frequency is higher than 1% in the Profile. b) The same procedure is applied on MGS clustered at the genus level in $H^{(mgs,nmf)}$. Taxonomic levels are genus, class and phyla. c) We display for each dataset the weights distribution in $W^{(AFT)}_{i,PG,nmf}$ with log-scale violin plots. We can observe a very irregular pattern compared to the weights $W^{(AFT)}_{i}$ obtained with AFT profiling, with profiles that are absent in certain datasets (e.g. W2 in the *hmp*2 dataset, or W1 in the *Parkinson* dataset). d) When BMI is available, samples are displayed in the $W1_{PG,nmf}$-$W2_{PG,nmf}$ space, colored by BMI. 95% confidence ellipse are indicated for BMI lower and higher than 35 (class 2, severe obesity threshold). e) Obesity status. Boxplot of $W1_{PG,nmf}$ and $W2_{PG,nmf}$ levels structured by obesity status. We can observe that the switch between W1 and W2 observed in AFT profiling for higher obesity classes (see Fig. 4a) is not recovered. Instead, a total disappearance of $W1_{PG,nmf}$ is observed, together with small or non-significant shifts in $W2_{PG,nmf}$ for strong obesity (MW = Mann-Whitney test). f) CD and healthy (N) dysbiotic (dys.) and not-dysbiotic(Not_Dys.) samples are displayed in a ternary plot in the $W1_{PG,nmf}-W2_{PG,nmf}-W3_{PG,nmf}$ space. g) Boxplot of the $W1_{PG,nmf}$, $W2_{PG,nmf}$ and $W3_{PG,nmf}$ levels, structured by dysbiotic and CD status. The pattern observed in Fig. 6d is not recovered. In particular, contrary to *W*2 and *W*3, $W2_{PG,nmf}$ and $W3_{PG,nmf}$ do not allow to finely discriminate between dysbiotic and not-dysbiotic CD samples.

**Additional file 10.** Metabolic exploration. A dynamical metabolic exploration is made available in html pages. Archives must be unzipped and the html file must be open in a web browser. • Profile AFT frequencies. HTML page sources *Within-profile-dynamical-map.zip*. • Metabolic shifts during CD and dysbiosis. HTML page sources *Metabolic-shifts-CD-dys-map.zip.*

**Additional file 11.** Dataset count matrices, Profile decomposition and metadata. The input data needed for the analysis are provided. • *Metadata.xlsx*: excel file containing the metadata of all the datasets used in the analysis. Field definition: – Sample_ID : internal ID. – ProjectID : study accession number. – SRA : SRA sample accession ID. – Patient_ID : internal patient ID. – Nationality, sex, age, BMI : patient nationality, sex, age and BMI. – Diagnosis : internal diagnosis code. N = healthy, CD = Crohn’s disease, UC = Ulcerative colitis, Control = Control sample, ObCIII = class 3 obesity, ObCII = class 2 obesity, ObCI= class 1 obesity, OW = overweight (but not obese), UW = underweight, PD = Parkinson disease, HC = healthy control, Diab = diabetis, ankylosing_spondylitis = ankylosing spondylitis. – Dysbiosis_index : dysbiosis index computed from HMP2 samples (see material and methods), Is_dysbiotic = above or under dysbiotic threshold (see material and methods). – Study : internal study ID. – reference : doi of the related publication. – alias : internal alias of the sample (HMP2 dataset only). – enterotype : sample enterotypes (metacardis dataset only). – Statin : statin intake (metacardis dataset only). – Bristol : Bristol score (metacardis dataset only). – Diet : diet taken by the patient (control or Mediterranean diet, med diet dataset only). – Timepoint : baseline, 4_weeks, 8_weeks (med_diet dataset only). • *W.xlsx*: weight matrix for the different datasets. • *X_AFT.xlsx*: AFT count matrix for the different datasets. The header indicates the AFT names as in Table 2. • *X_mgs.xlsx*: MGS count matrix at the genus level (train dataset only). • *X_pg.xlsx*: Prevalent genomes count matrix for the different datasets. The first sheet indicates the NCBI taxonomy ID and the name of the 203 prevalent genomes included in the study. • *Genome_list.xlsx*: list of the 203 genomes included in the study. • *H.xlsx*: matrices $H^{(AFT)}, H^{(PG)}, H^{(mgs)}, H^{(AFT, mtx)}, H^{(PG, nmf)}$ and $H^{(mgs, nmf)}$. • *W_NMF_taxonomy.xlsx*: weight matrix for the different datasets, for taxonomy-only NMF. • *List_of_Reactions.xlsx*: List of reactions as indicated in Fig. 1 and Table 2, with complete aggregated biochemical reaction, and reactant names. • *F.xlsx*: matrix of metabolic constraints used during NMF.

**Additional file 12.** Supplementary materials. This document recapitulates additional precisions on the material and methods involved in this study.

## Data Availability

All data and codes are provided in the supplementary materials and the “Methods”section.
