# Supplementary figures and images for: Four functional profiles for fibre and mucin metabolism in the human gut microbiome
Source: Microbiome. 2023 Oct 20;11:231. doi: 10.1186/s40168-023-01667-y (PMC10588041; doi:10.1186/s40168-023-01667-y)

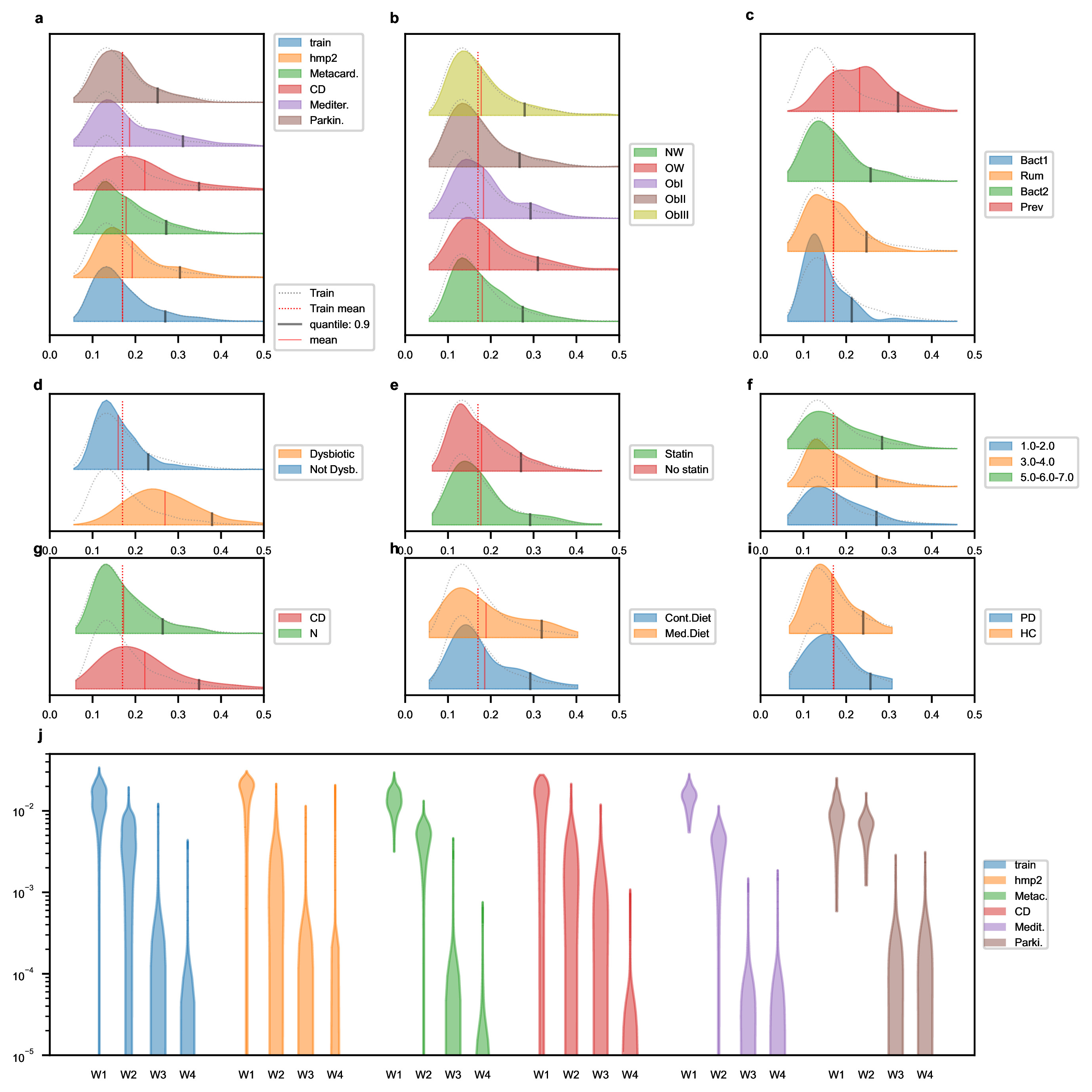

Supplement: Supplementary file 2 — Additional file 1: Figure S1. AFT reconstruction error distribution and weight distribution. The relative reconstruction error distribution among samples defined as $\left\| X^{(AFT)}_{g,i} - W^{(AFT)}_{g,i}H^{(AFT)}\right\| \left/\left\| X^{(AFT)}_{g,i}\right\|\right.$Xg,i(AFT)-Wg,i(AFT)H(AFT)Xg,i(AFT) is displayed, and structured according to the different groups g encountered along the study i.e. a) datasets, b) obesity status, c) enterotypes, d) dysbiotic status, e) statin intake, f) Bristol score, g) Crohn disease status, h) Mediterranean or control diet and i) parkinson disease. For comparison, the distribution observed in the train dataset is displayed in all graphs (gray dash lines), together with its mean relative reconstruction error (red dashed line). The mean and quantile 90% of each distribution are displayed with the vertical red and black lines. We can see that the relative reconstruction error distributions are very homogenenous along every structuring variables, except for dysbiotic and CD samples and Prevotela enterotypes, where relative reconstruction error is increased, but keeping the 95% quantile under 44% of reconstruction error. All together, the functional profiles allow to reconstruct the large majority of external samples with a level of accuracy comparable to the training dataset reconstruction, with a higher bias for dysbiotic, CD and Prevotela samples. j) The distribution of the weights $W^{(AFT)}_{i}$Wi(AFT) are displayed for each dataset, with violin plots [file 40168_2023_1667_MOESM1_ESM.jpeg]

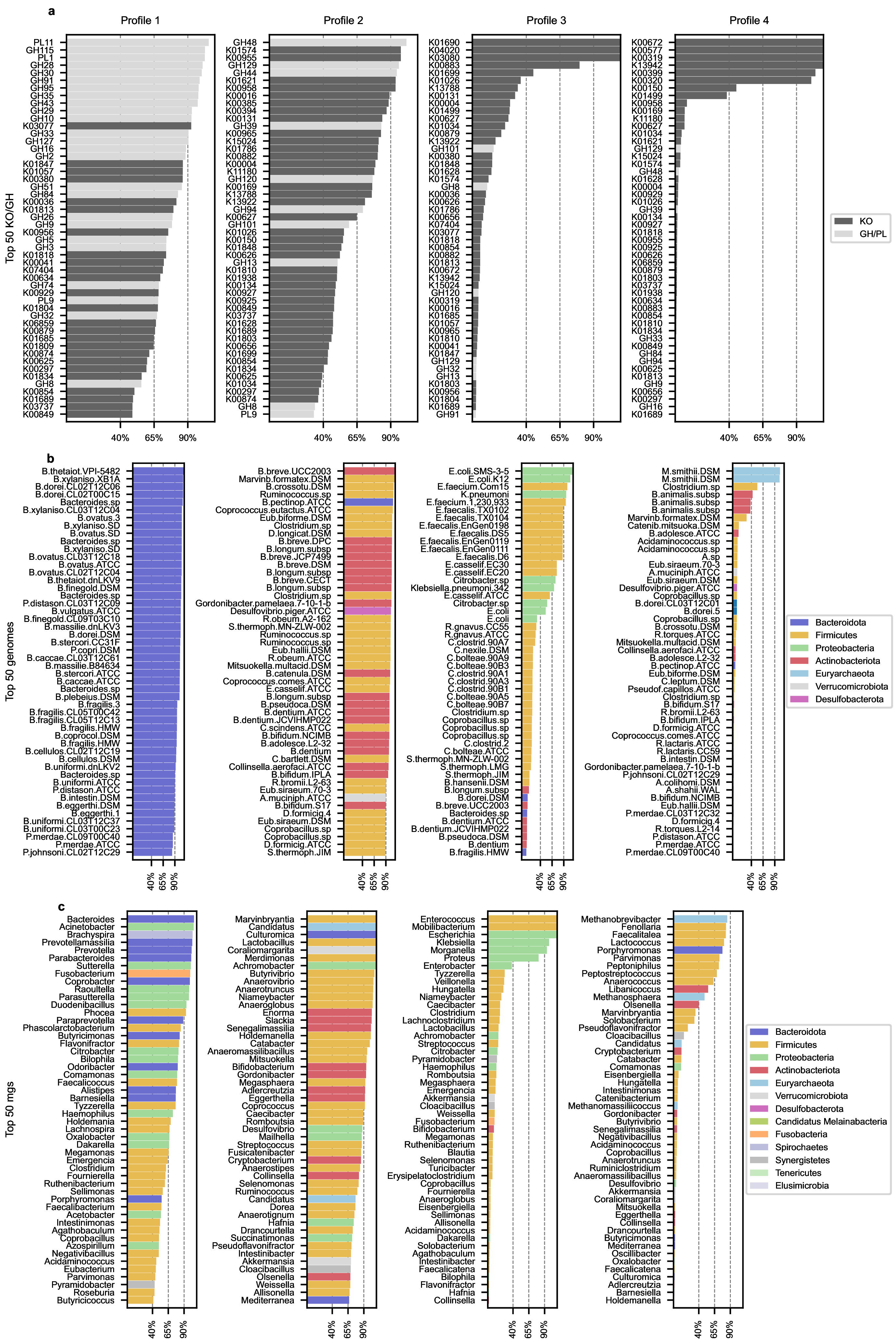

Supplement: Supplementary file 3 — Additional file 2: Figure S2. Top functional and taxonomic profiles contribution to metagenome. The top 50 relative profiles contribution to a) AFTs b) PGs and c) MGS-derived genus reconstruction are displayed. Namely, we compute for Profile i and AFT or genome j the profiles contribution $\bar{W}^{(AFT)}_{train,i}H^{(AFT)}_j \left/\bar{X}^{(AFT)}_{train,j}\right.$W¯train,i(AFT)Hj(AFT)X¯train,j(AFT) where $\bar{W}^{(AFT)}_{train}$W¯train(AFT) and $\bar{X}^{(AFT)}_{train}$X¯train(AFT) are averaged among the training samples. Then, contributions are sorted and top 50 contributions are kept and colorcoded by KO or GH for AFT, and phylum for PGs and MGS clustered by genus. Profile 1 is characterized by an over-representation of GH and Bacteroidetes, while Profile 2 is characterized by more KOs, and Firmicutes and Actinobacteriota. [file 40168_2023_1667_MOESM2_ESM.jpeg]

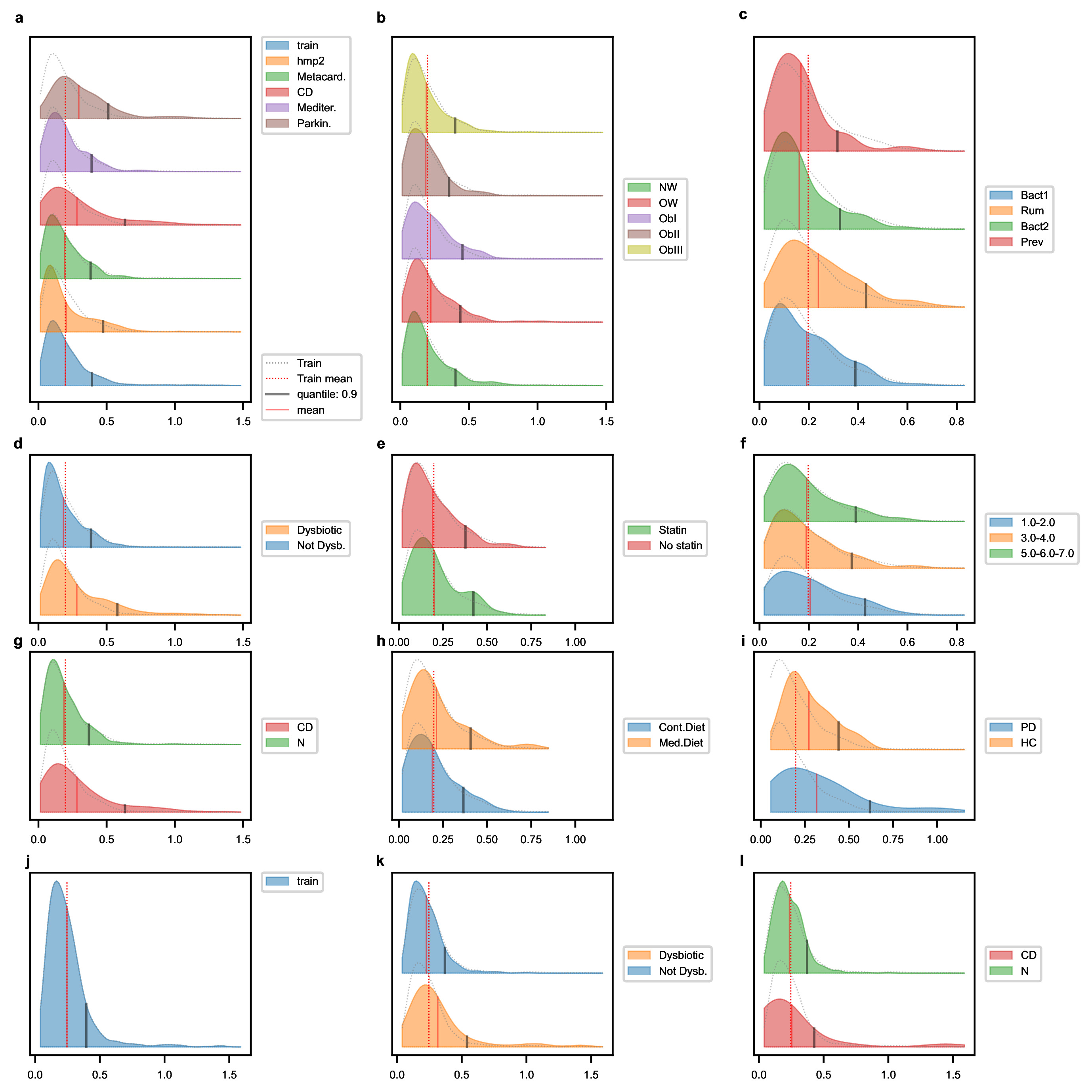

Supplement: Supplementary file 4 — Additional file 3: Figure S3. Phyla reconstruction error distribution when reconstructing the PG counts. The phyla relative reconstruction error distribution among samples defined as $\left\| \left(X^{(PG)}_{i} - W^{(AFT)}_{i}H^{(PG)}\right).A_{phyla}\right\| \left/\left\| X^{(PG)}_{i}.A_{phyla}\right\|\right.$Xi(PG)-Wi(AFT)H(PG).AphylaXi(PG).Aphyla is displayed, where $X^{(PG)}$X(PG) is the count matrix of the 203 representative genomes and $A_{phyla}$Aphyla is an allocation matrix of each genome to its phyla, and structured according to the different classes encountered along the study, i.e. a) datasets, b) obesity status, c) enterotypes, d) dysbiotic status, e) statin intake, f) Bristol score, g) Chron disease status, h) Mediterranean or control diet and i) parkinson disease. For comparison, the distribution observed in the train dataset is displayed in all graphs (gray dash lines), together with its mean relative reconstruction error (red dashed line). The mean and quantile 90% of each distribution are displayed with the vertical red and black lines. We can see that the relative reconstruction error distributions of the phyla are very homogenenous along every structuring variables, except for dysbiotic, CD and Parkinson disease samples, where r [file 40168_2023_1667_MOESM3_ESM.jpeg]

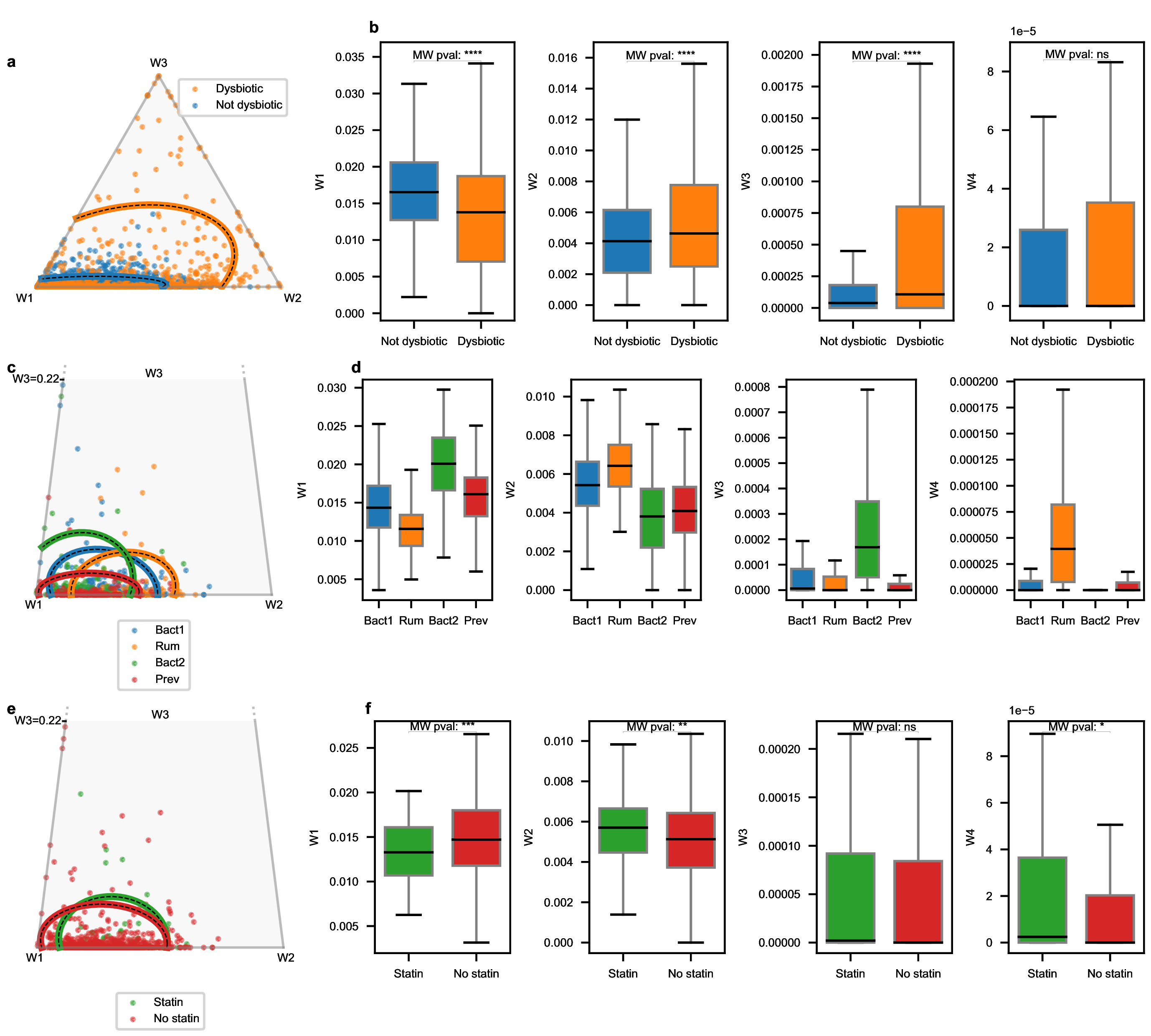

Supplement: Supplementary file 5 — Additional file 4: Figure S4. Characterization of dysbiosis, enterotypes and statin related samples. a) Dysbiosis. Ternary plot in the $W_1-W_2-W_3$W1-W2-W3 space of samples colored by dysbiotic status. We also display the 95% confidence area for each category (colored line). b) Boxplot of $W^{(AFT)}_{1}$W1(AFT), $W^{(AFT)}_{2}$W2(AFT), $W^{(AFT)}_{3}$W3(AFT) and $W_4^{(AFT)}$W4(AFT) ﻿levels, structured by dysbiotic status. We can observe that dysbiotic samples are characterized by significantly lower $W^{(AFT)}_{1}$W1(AFT), highe [file 40168_2023_1667_MOESM4_ESM.jpeg]

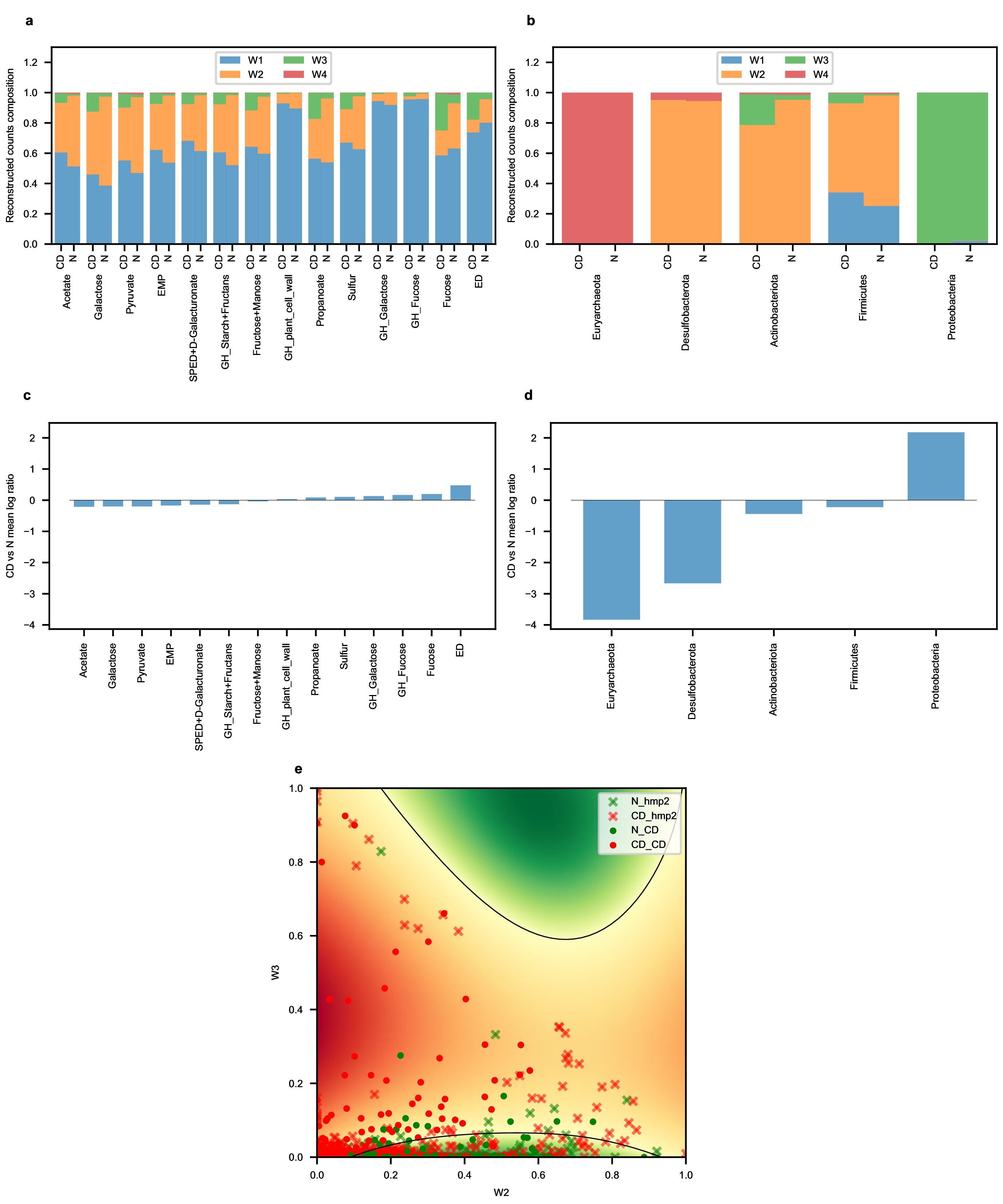

Supplement: Supplementary file 6 — Additional file 5: Figure S5. CD-related profiles characterization. a) Functional differential analysis between CD and healthy samples (N). Average profile contribution in the significantly different functional module frequencies for CD and N groups. Functional modules are defined in fig. 1. a. We averaged the $L_1$L1 normalized $W^{(AFT)}$W(AFT) (resp. $X^{(AFT)}$X(AFT)) for the CD and N groups of the train dataset, noted $\bar{W}^{(AFT)}_{train,L_1,g}$W¯train,L1,g(AFT) (resp. $\bar{X}^{(AFT)}_{train,L_1,g}$X¯train,L1,g(AFT)) for $g=CD$ [file 40168_2023_1667_MOESM5_ESM.jpeg]

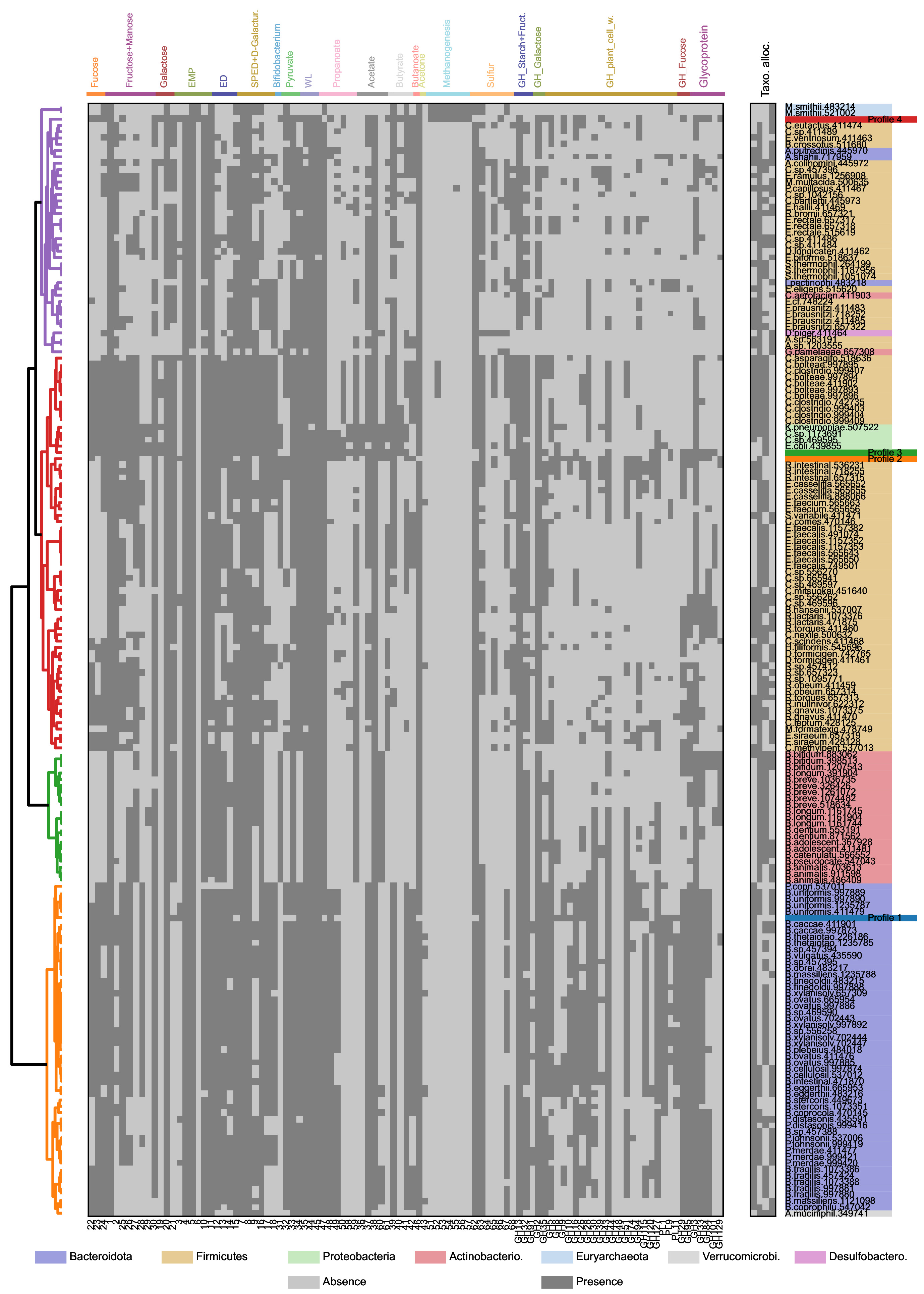

Supplement: Supplementary file 7 — Additional file 6: Figure S6. Prevalent genomes functional profiling. Selected AFTs are annotated in the prevalent genomes and presence/absence is displayed (middle panel), sorted by functional blocks (top). The genome names are indicated (short name and NCBI ID, right panel), colorcoded by phylum, and the the taxonomic allocation of the genomes in profiles is indicated by the presence/absence matrix in $H^{(PG)}$H(PG) (right panel, taxo. alloc. Profile i is the i-th column of this matrix). The 4 profiles are added to the genome list and displayed with presence/absence tags (a KO is assumed present in the Profile if its frequence is higher than $1e-3$1e-3). Hierarchical clustering is performed ($k=4$k=4 clusters), based on pairwise-Jaccard distance computed on AFT presence/absence matrix (corresponding dendogram in the left panel), and genomes are sorted accordingly in the middle and right panels. We note that the 4 profiles are clustered at the same time than the genomes. Bacteroidetes and Actinobacteria are gathered into their own cluster (orange and green clusters), whereas Firmicutes are splitted in two clusters: the main part is clustered with Proteobacteria (red), while the others are clustered with less prevalent phyla such as Desulfobactero [file 40168_2023_1667_MOESM6_ESM.jpeg]

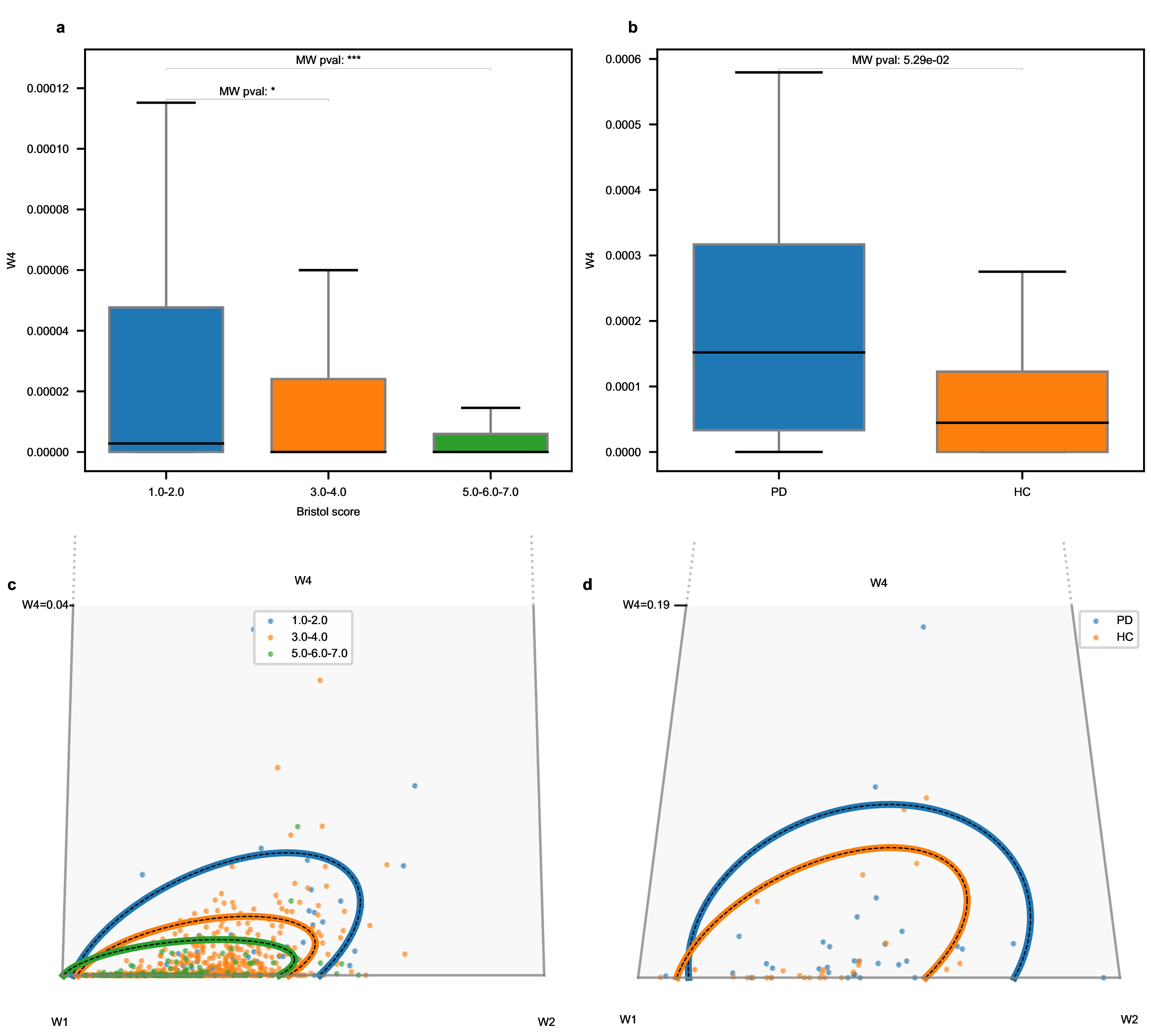

Supplement: Supplementary file 8 — Additional file 7: Figure S7. Profile 4 association with Bristol score and Parkinson’s disease. a) Bristol score. Boxplot of $W^{(AFT)}_{4}$W4(AFT) levels, structured by Bristol stool score. b) Parkinson’s disease. Boxplot of $W^{(AFT)}_{4}$W4(AFT) levels in PD and healthy control samples. We can observe that the significance of the difference between groups is slight ($p=5.3e-2$p=5.3e-2, MW test) c) Ternary plot in the $W_1-W_2-W_4$W1-W2-W4 space, colored by Bristol stool score. 95% confidence ellipses of each class are displayed. d) Ternary plot in the $W_1-W_2-W_4$W1-W2-W4 space of PD and healthy control samples. 95% confidence ellipses of each class are displayed. [file 40168_2023_1667_MOESM7_ESM.jpeg]

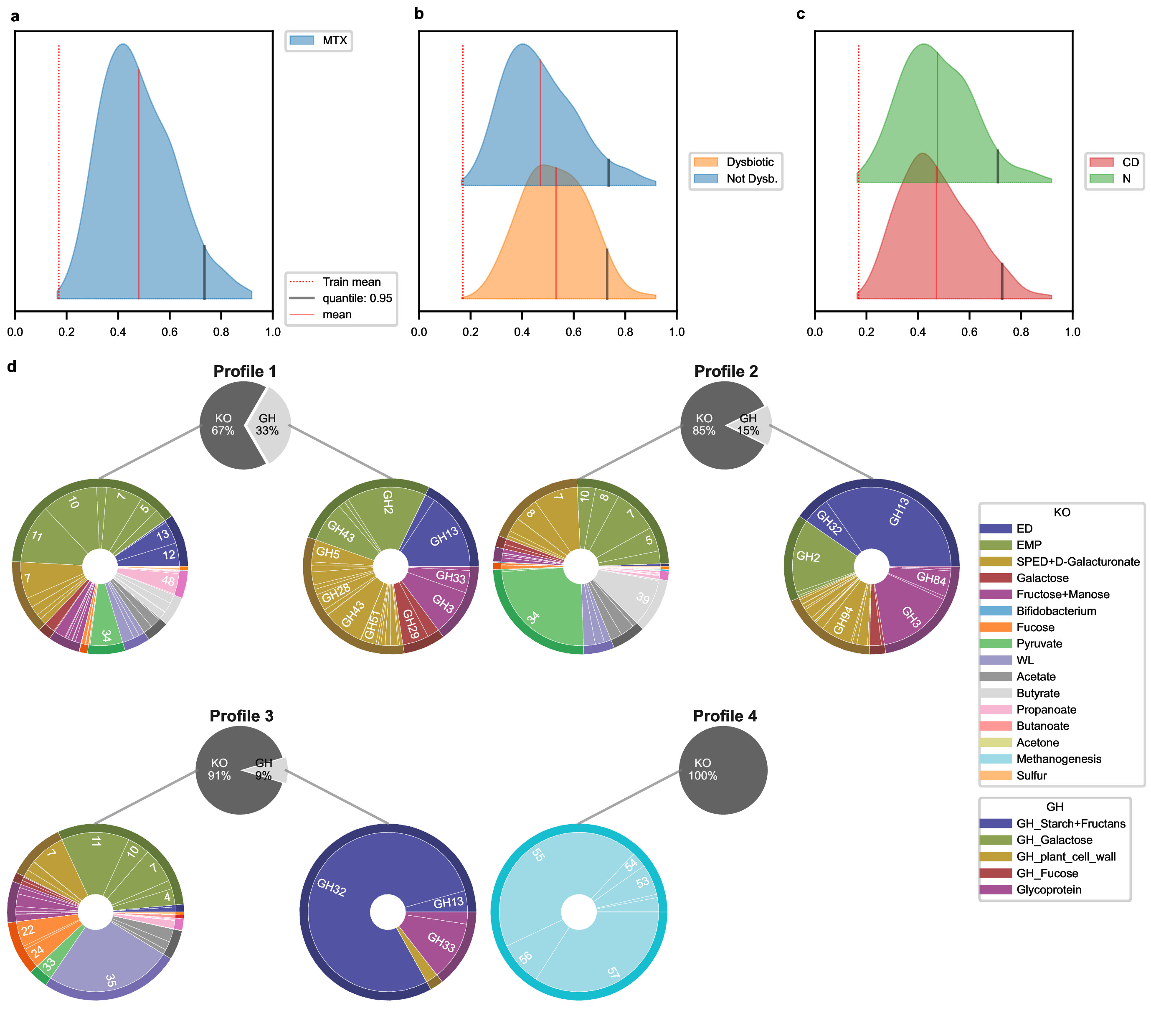

Supplement: Supplementary file 9 — ﻿Additional file 8: Figure S8. Expression profiling by searching for transcripts co-varying with AFT profiles in metatranscriptomics data. a) Reconstruction error distribution. Metatranscriptomics data are acquired from the hmp2 database, and an AFT expression count matrix $X^{(AFT,mtx)}$X(AFT,mtx) is assembled. Expression profiles are constructed by computing Hmtx such that $X^{(AFT,mtx)} \simeq W^{(AFT)}_{H^{(AFT,mtx)}}$X(AFT,mtx)≃WH(AFT,mtx)(AFT) by NNLS, i.e. by searching for AFT expressions that co-varies with the AFT profiles $H^{(AFT)}$H(AFT). Relative reconstruction error distribution among samples defined as $\left.\left\| X^{(AFT,mtx)}{g,i} - W^{(AFT)}_{g,i}H^{(AFT,mtx)}\right\| \right/\left\| X^{(AFT,mtx)}{g,i}\right\|$X(AFT,mtx)g,i-Wg,i(AFT)H(AFT,mtx)X(AFT,mtx)g,i is displayed, and structured according to the different groups g encountered in the hmp2 dataset i.e. dysbiotic, non dysbiotic, Chron’s disease and healthy patients. The vertical dotted red line show the average reconstruction error on A [file 40168_2023_1667_MOESM8_ESM.jpeg]

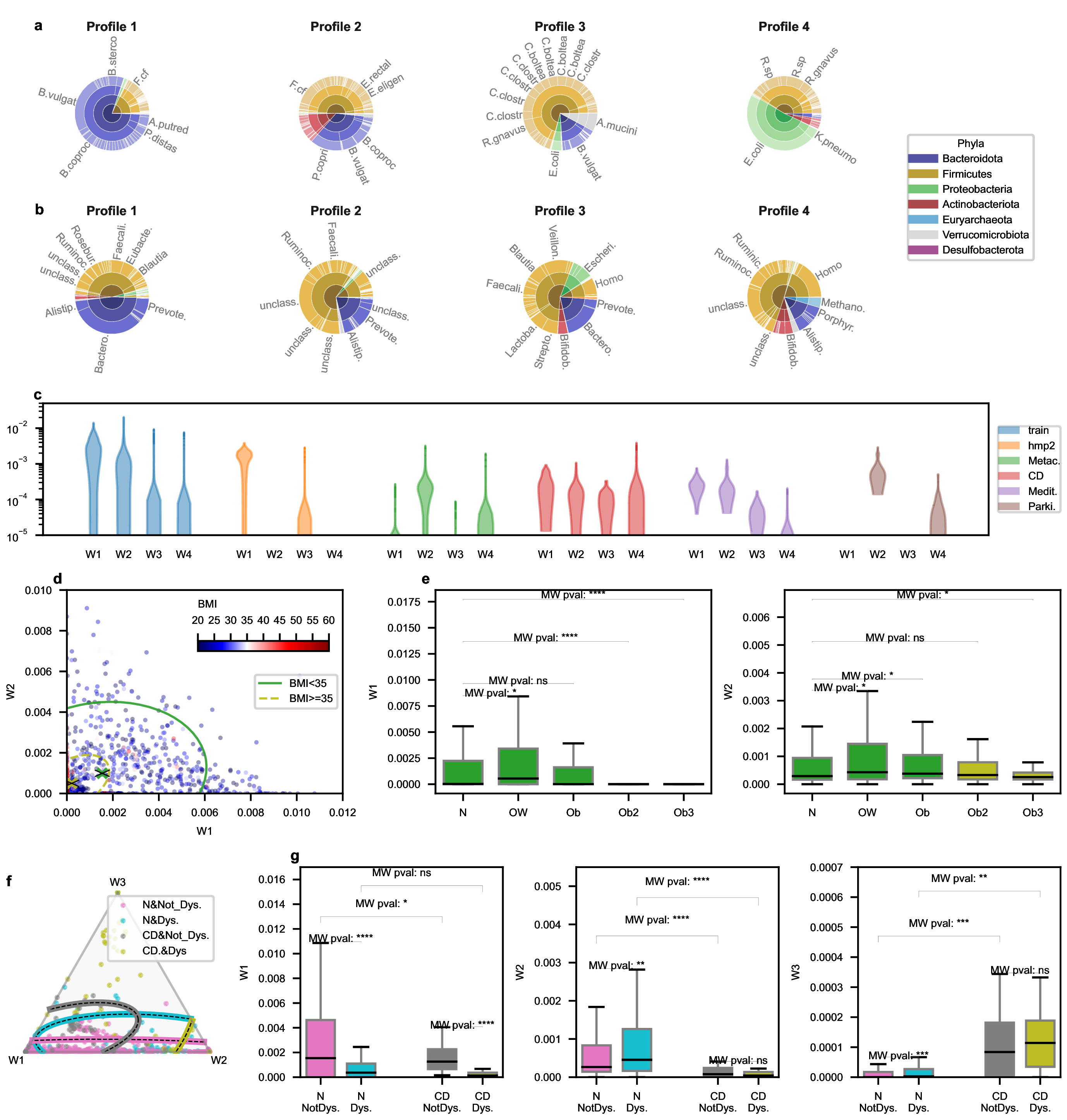

Supplement: Supplementary file 10 — Additional file 9: Figure S9. Taxonomy-only profiling of the metagenomes. We performed a NMF on the $X^{(PG)}$X(PG) and $X^{(mgs)}$X(mgs) taxonomy count matrices to recover the couples $\left(W^{(mgs,nmf)}_{train},H^{(PG,nmf)}\right)$Wtrain(mgs,nmf),H(PG,nmf) and $\left(W^{(mgs,nmf)}_{train},H^{(mgs,nmf)}\right)$Wtrain(mgs,nmf),H(mgs,nmf) so that $X^{(PG)}_{train} \simeq W^{(PG,nmf)}_{train} H^{(PG,nmf)}$Xtrain(PG)≃Wtrain(PG,nmf)H(PG,nmf) and $X^{(mgs)}_{train} \simeq W^{(mgs,nmf)}_{train} H^{(mgs,nmf)}$Xtrain(mgs)≃Wtrain(mgs,nmf) [file 40168_2023_1667_MOESM9_ESM.jpeg]
