# Supplementary material for: Four functional profiles for fibre and mucin metabolism in the human gut microbiome
Source: Microbiome. 2023 Oct 20;11:231. doi: 10.1186/s40168-023-01667-y (PMC10588041; doi:10.1186/s40168-023-01667-y)
Supplement: Supplementary file 13 — Additional file 12. Supplementary materials. This document recapitulates additional precisions on the material and methods involved in this study. [file 40168_2023_1667_MOESM12_ESM.pdf]

RESEARCH

Four functional profiles for fiber and mucin metabolism in the human gut microbiome – Supplementary material

Simon Labarthe[1,2,3*], Sandra Plancade[1,4], Sebastien Raguideau[1,5], Florian Plaza-Onate[6], Emmanuelle Le Chatelier[6], Beatrice Laroche[1,7] and Marion Leclerc[8,9]

*Correspondence:
simon.labarthe@inrae.fr
[1]Université Paris-Saclay, INRAE, MaIAGE, 78350 Jouy-en-Josas, France
[2]Univ. Bordeaux, INRAE, BIOGECO, 33610 Cestas, France
[3]Inria, INRAE, Pléiade, 33400 Talence, France
Full list of author information is available at the end of the article

Abstract

Background: With the emergence of metagenomic data, multiple links between the gut microbiome and the host health have been shown. Deciphering these complex interactions require evolved analysis methods focusing on the microbial ecosystem functions. Despite the fact that host or diet-derived fibres are the most abundant nutrients available in the gut, the presence of distinct functional traits regarding fibre and mucin hydrolysis, fermentation and hydrogenotrophic processes has never been investigated.

Results: After manually selecting 91 KEGG orthologies and 33 glycoside hydrolases further aggregated in 101 functional descriptors representative of fibre and mucin degradation pathways in the gut microbiome, we used nonnegative matrix factorization to mine metagenomic datasets. Four distinct metabolic profiles were further identified on a training set of 1153 samples and thoroughly validated on a large database of 2571 unseen samples from 5 external metagenomic cohorts. Profiles 1 and 2 are the main contributors to the fibre-degradation-related metagenome: they present contrasted involvement in fibre degradation and sugar metabolism and are differentially linked to dysbiosis, metabolic disease and inflammation. Profile 1 takes over Profile 2 in healthy samples, and unbalance of these profiles characterize dysbiotic samples. Furthermore, high fibre diet favours a healthy balance between Profiles 1 and Profile 2. Profile 3 takes over Profile 2 during Crohn's disease, inducing functional reorientations towards unusual metabolism such as fucose and H2S degradation or propionate, acetone and butanediol production. Profile 4 gathers under-represented functions, like methanogenesis. Two taxonomic makes up of the profiles were investigated, using either the covariation of 202 prevalent genomes or metagenomic species, both providing consistent results in line with their functional characteristics. This taxonomic characterization showed that Profiles 1 and 2 were respectively mainly composed of bacteria from the phyla *Bacteroidetes* and *Firmicutes* while Profile 3 is representative of *Proteobacteria* and Profile 4 of methanogens.

Conclusions: Integrating anaerobic microbiology knowledge with statistical learning can narrow down the metagenomic analysis to investigate functional profiles. Applying this approach to fibre degradation in the gut ended with 4 distinct functional profiles that can be easily monitored as markers of diet, dysbiosis, inflammation and disease.

Keywords: metagenomics; NMF; functional profiling; statistical learning

Additional methodological details

We further detail here methodological steps of the method.

KO selection

We recall here the method presented in [1]. From the pathways selected, a list of putative reactions was compiled. For each reaction, a KEGG database reaction entry was retrieved. Since each reaction is catalyzed by enzymes linked to KO, a list of putative KO was obtained. The manual curation of the KO candidates followed the rules: (1) a KO not found in the IGC annotation was ignored, (2) a reaction that was not linked to a KO was ignored, (3) when an enzyme from a KO could catalyze more than one reaction, because we could not accurately link a KO frequency to a target reaction, all the KO associated to the target reaction were ignored. Exceptions were made regarding key-reactions. Multiple KO associated to a unique reaction were kept since they correspond to different enzymes catalyzing the same chemical reaction in different species (L-ribulose to G-Gly 3 Phosphate, Acetyl-CoA to Acetyl-Phosphate, Lactate to propionate) or different subunits of the same enzyme, such as K01034 and K01035. Reactions associated with microorganisms unlikely present in the gut microbiomes, such as aerobes from soil, halophilic extremophiles, were excluded. Reactions associated to micro-aerophilic or facultative anaerobes were kept. KO from very low abundant microorganism from the gut microbiome were kept.

Rationale and assemblage for the constraint matrix F

The metabolites in the model were parted between those that were known to be extracellular (gathered in a set noted E, and displayed with bold box in Fig. 1.A) and the others (not known to be extracellular, gathered in a set NE, and gathered with gray box in Fig.1.A). For a metabolite $m \in NE$, we considered all the reactions in our list that could produce m and gathered all the associated traits in a set called P_m. In the same way, all the traits involved in reactions that could consume m were gathered in a set called C_m. Each profile (line of H) was constrained so that the total sum of producing and consuming can not be simultaneously null. Namely, for profile number l, we state that

$$\sum_{j \in C_m} H_{lj} \leq \alpha_m^+ \left(\sum_{j \in P_m} H_{lj} \right) \tag{1}$$

$$\sum_{j \in P_m} H_{lj} \leq \alpha_m^- \left(\sum_{j \in C_m} H_{lj} \right) \tag{2}$$

for given coefficients α_m^+ and α_m^- to be defined.

The rationale of these constraints is to prevent the accumulation of intracellular compounds, and therefore if there is a potential in the profile for producing a metabolite, the same profile should also carry a functional potential for using it, and conversely. The bounds α_m^+ and α_m^- were derived from the analysis of the 190 prevalent genomes. In a nutshell, metabolites were constrained only if bounds α_m^+ or α_m^- could be found such that Eq. 1 or 2 are satisfied for more than 95% of the

190 genomes when replacing the H_{lj} by the corresponding KO frequencies in each genomes. Moreover, a security margin was taken on the values of α_m^+ and α_m^- to account for a possible discrepancy between the 190 representative genomes and the full microbiota. See [1] for extended justification of these constraints and a study of their impact on a toy model and a real case study.

Hyper-parameter selection procedure

Following [1], we follow a three-step hyperparameter selection procedure based on (1) a reconstruction error criteria, (2) a bi-cross validation and (3) a criteria based on the stability of the inferred H.

Reconstruction error. The relative reconstruction error criteria measures the proportion of information recovered by the NMF decomposition

$$C_{rec.err.} = \frac{\|X_{train}^{(AFT)} - W_{train}H^{(AFT)}\|_F}{\|X_{train}^{(AFT)}\|_F}.$$

This criteria mechanically decreases with the number of profiles k, thus a slope discontinuity on the graph is searched for, indicating that additional profiles carry less information and mainly approximate noise.

Bi-cross validation criteria. Starting from $X_{train}^{(AFT)}$ of size $n_s = 1126$ samples times $n_{AFT} = 101$ AFTs, we set a 5-fold random splitting l_s and l_{AFT} of respectively the set of samples and AFTs. Taking $I \in l_s$ and $J \in l_{AFT}$ two index subsets taken from the 5-fold splitting, we note

$$\begin{aligned} X_{11}^{IJ} &= (X_{train\ kl}^{(AFT)})_{k\in I, l\in J}, \quad X_{12}^{IJ} = (X_{train\ kl}^{(AFT)})_{k\in I, l\notin J}, \\ X_{21}^{IJ} &= (X_{train\ kl}^{(AFT)})_{k\notin I, l\in J}, \quad X_{22}^{IJ} = (X_{train\ kl}^{(AFT)})_{k\notin I, l\notin J}, \end{aligned}$$

We then note W_1^{IJ}, H_1^{IJ} the solution of the NMF decomposition of X_{11}^{IJ}

$$(W_1^{IJ}, H_1^{IJ}) = \underset{\substack{W \geq 0 \\ H \geq 0}}{\arg\min} \|(X_{11}^{IJ} - WH)D^{-1}\|_F^2 + \alpha\left(\|W\|_F^2 + \|HD^{-1}\|_{1,2}^2\right) \quad (3)$$

We note that this NMF is unconstrained since the undersampling breaks up the structure of the constraints.

We note W_2^{IJ} and H_2^{IJ} the respective non-negative least-square regression of X_{21}^{IJ} and X_{12}^{IJ} of the (unconstrained) problems

$$W_2^{IJ} = \underset{W \geq 0}{\arg\min} \|(X_{21}^{IJ} - WH_1)D^{-1}\|_F^2 + \alpha\left(\|W\|_F^2\right) \quad (4)$$

and

$$H_2^{IJ} = \underset{H \geq 0}{\arg\min} \|(X_{12}^{IJ} - W_1H)D^{-1}\|_F^2 + \alpha\left(\|HD^{-1}\|_{1,2}^2\right) \quad (5)$$

The criteria is the average over I and J of the relative reconstruction error of X_{22}^{IJ}

$$C_{bi-cross} = \frac{1}{|l_s| \times |l_{AFT}|} \sum_{I \in l_s} \sum_{J \in l_{AFT}} \frac{\|X_{22}^{IJ} - W_2^{IJ} H_2^{IJ}\|_F}{\|X_{22}^{IJ}\|_F}$$

Stability. After splitting the training set in two balanced random subsets X_1^J and X_2^J of $X_{train}^{(AFT)}$, $1 \leq J \leq 20$ being the index of the splitting in 20 splitting repetition, a constrained NMF is performed on X_1^J and X_2^J to get (W_1^J, H_1^J) and (W_2^J, H_2^J). To assess the similarity between profiles, we compute the similarity matrix for $I = 1, 2$ of dimension $n_{AFT} \times n_{AFT}$

$$S_{I\,lm}^J = \frac{\sum_{i=1}^k H_{I\,il}^J H_{I\,im}^J}{\left(\sum_{i=1}^k {H_{I\,il}^J}^2\right)^{1/2} \left(\sum_{i=1}^k {H_{I\,im}^J}^2\right)^{1/2}}, \qquad \text{for } 1 \leq l, m \leq N_{AFT}$$

The criteria is finally

$$C_{stability} = 1 - \frac{1}{20\sqrt{n_{AFT}(n_{AFT} - 1)}} \sum_{J=1}^{20} \|S_1^J - S_2^J\|_F$$

Next, we compute the different criteria in a grid with $\alpha \in \{0.001, 0.01, 0.03162, 0.1, 1\}$ and $k \in \{2, 4, 6, 8, 11\}$ (see 1) and selected $\alpha = 0.03162$, providing the minimal value for the bi-cross validation and equivalent values for the other criteria. Next, in order to have a deeper accuracy on the selection ot the number of profiles, we computed the criteria for $\alpha = 0.03162$ and $k = 2, \cdots, 12$. We selected $k = 4$ due to the clear slope break for the stability criteria and un-degraded accuracy for the other criteria.

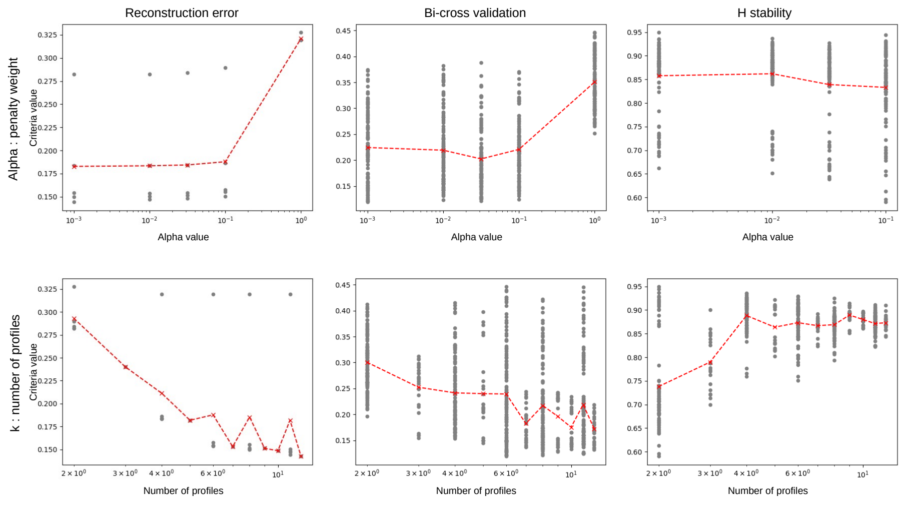

Figure 1 Hyperparameter selection. The results of the hyperparameter selection procedure are displayed for α selection (upper panel) and the number k of profiles (lower panel) for the reconstruction error, the bi-cross validation and the H stability criteria. This procedure leads to select $k = 4$ profiles and a value of $\alpha = 0.03162$

Author details
[1]Université Paris-Saclay, INRAE, MaIAGE, 78350 Jouy-en-Josas, France. [2]Univ. Bordeaux, INRAE, BIOGECO, 33610 Cestas, France. [3]Inria, INRAE, Pléiade, 33400 Talence, France. [4]INRAE, MIAT, 31 Toulouse, France. [5]Affiliation Sebastien, , Warwick, UK. [6]Université Paris-Saclay, INRAE, MGP, 78350 Jouy-en-Josas, France. [7]Inria, INRAE, Musca, 91120 Palaiseau, France. [8]Université Paris-Saclay, INRAE, Micalis, 78350 Jouy-en-Josas, France. [9]Pendulum Therapeutics, San Francisco, USA.

References

1. Raguideau, S., Plancade, S., Pons, N., Leclerc, M., Laroche, B.: Inferring aggregated functional traits from metagenomic data using constrained non-negative matrix factorization: Application to fiber degradation in the human gut microbiota. PLoS computational biology **12**(12), 1005252 (2016)
