# Supplementary material for: Four functional profiles for fibre and mucin metabolism in the human gut microbiome
Source: Microbiome. 2023 Oct 20;11:231. doi: 10.1186/s40168-023-01667-y (PMC10588041; doi:10.1186/s40168-023-01667-y)
Supplement: Supplementary file 11 — Additional file 10. Metabolic exploration. A dynamical metabolic exploration is made available in html pages. Archives must be unzipped and the html file must be open in a web browser. • Profile AFT frequencies. HTML page sources Within-profile-dynamical-map.zip. • Metabolic shifts during CD and dysbiosis. HTML page sources Metabolic-shifts-CD-dys-map.zip. [file 40168_2023_1667_MOESM10_ESM.zip › Metabolic-shifts-CD-dys-map.zip!/CD_characterization/slider_CD_N.html]

 


N&Undys

N&Dys


CD&Undys

CD&Dys

Undysbiotic healthy patient

**Variation of profile contributions in healthy vs CD, and dysbiotic vs not dysbiotic samples.** The metabolic network of fibre degradation is displayed, and profile contribution in GH/PL and KO counts is colour coded on the corresponding arrows of the network. Profile contributions are displayed for healthy (N) and CD samples, and dysbiotic (Dys) and undysbiotic (Undys) samples. Namely, we compute CD and healthy average profiles weight  W  ‾ train,g(AFT) by averaging  W train(AFT) on the sample group g (N&Dys, N&Undys, CD&Dys, CD&Undys). Average AFT counts  X  ‾ train,g(AFT) are obtained in the same manner for each group. Then, average profile contribution for AFT j and profile i is computed with  W  ‾ train,g,i(AFT) H ij(AFT) X  ‾ train,g,j(AFT). The respective relative contribution of Profile 1, 2 and 3 is then mapped into a ternary colour map and displayed on the corresponding arrow or GH/PL box. Black arrows indicate AFT the main contribution of which is given by Profile 4. Arrow widths are proportional to AFT counts in  X  ‾ train,g(AFT). For N&undys graph, all the AFTs are represented (control situation). For the other graph, the AFTs that significantly changed compared to N&undys group (t-test and Benjamini Hochberg correction with $FDR < 0.05$) were filtered; we then ordered AFTs by compositional changes compared to N&Undys group (L2 difference on  W  ‾ train,g,i(AFT) H ij(AFT) X  ‾ train,g,j(AFT) computed on both groups) and kept the top 20 AFTs in order to highlight the main changes in microbiota composition.

Dysbiotic healthy patient

**Variation of profile contributions in healthy vs CD, and dysbiotic vs not dysbiotic samples.** The metabolic network of fibre degradation is displayed, and profile contribution in GH/PL and KO counts is colour coded on the corresponding arrows of the network. Profile contributions are displayed for healthy (N) and CD samples, and dysbiotic (Dys) and undysbiotic (Undys) samples. Namely, we compute CD and healthy average profiles weight  W  ‾ train,g(AFT) by averaging  W train(AFT) on the sample group g (N&Dys, N&Undys, CD&Dys, CD&Undys). Average AFT counts  X  ‾ train,g(AFT) are obtained in the same manner for each group. Then, average profile contribution for AFT j and profile i is computed with  W  ‾ train,g,i(AFT) H ij(AFT) X  ‾ train,g,j(AFT). The respective relative contribution of Profile 1, 2 and 3 is then mapped into a ternary colour map and displayed on the corresponding arrow or GH/PL box. Black arrows indicate AFT the main contribution of which is given by Profile 4. Arrow widths are proportional to AFT counts in  X  ‾ train,g(AFT). For N&undys graph, all the AFTs are represented (control situation). For the other graph, the AFTs that significantly changed compared to N&undys group (t-test and Benjamini Hochberg correction with $FDR < 0.05$) were filtered; we then ordered AFTs by compositional changes compared to N&Undys group (L2 difference on  W  ‾ train,g,i(AFT) H ij(AFT) X  ‾ train,g,j(AFT) computed on both groups) and kept the top 20 AFTs in order to highlight the main changes in microbiota composition.

Undysbiotic CD patient

**Variation of profile contributions in healthy vs CD, and dysbiotic vs not dysbiotic samples.** The metabolic network of fibre degradation is displayed, and profile contribution in GH/PL and KO counts is colour coded on the corresponding arrows of the network. Profile contributions are displayed for healthy (N) and CD samples, and dysbiotic (Dys) and undysbiotic (Undys) samples. Namely, we compute CD and healthy average profiles weight  W  ‾ train,g(AFT) by averaging  W train(AFT) on the sample group g (N&Dys, N&Undys, CD&Dys, CD&Undys). Average AFT counts  X  ‾ train,g(AFT) are obtained in the same manner for each group. Then, average profile contribution for AFT j and profile i is computed with  W  ‾ train,g,i(AFT) H ij(AFT) X  ‾ train,g,j(AFT). The respective relative contribution of Profile 1, 2 and 3 is then mapped into a ternary colour map and displayed on the corresponding arrow or GH/PL box. Black arrows indicate AFT the main contribution of which is given by Profile 4. Arrow widths are proportional to AFT counts in  X  ‾ train,g(AFT). For N&undys graph, all the AFTs are represented (control situation). For the other graph, the AFTs that significantly changed compared to N&undys group (t-test and Benjamini Hochberg correction with $FDR < 0.05$) were filtered; we then ordered AFTs by compositional changes compared to N&Undys group (L2 difference on  W  ‾ train,g,i(AFT) H ij(AFT) X  ‾ train,g,j(AFT) computed on both groups) and kept the top 20 AFTs in order to highlight the main changes in microbiota composition.

Dysbiotic CD patient

**Variation of profile contributions in healthy vs CD, and dysbiotic vs not dysbiotic samples.** The metabolic network of fibre degradation is displayed, and profile contribution in GH/PL and KO counts is colour coded on the corresponding arrows of the network. Profile contributions are displayed for healthy (N) and CD samples, and dysbiotic (Dys) and undysbiotic (Undys) samples. Namely, we compute CD and healthy average profiles weight  W  ‾ train,g(AFT) by averaging  W train(AFT) on the sample group g (N&Dys, N&Undys, CD&Dys, CD&Undys). Average AFT counts  X  ‾ train,g(AFT) are obtained in the same manner for each group. Then, average profile contribution for AFT j and profile i is computed with  W  ‾ train,g,i(AFT) H ij(AFT) X  ‾ train,g,j(AFT). The respective relative contribution of Profile 1, 2 and 3 is then mapped into a ternary colour map and displayed on the corresponding arrow or GH/PL box. Black arrows indicate AFT the main contribution of which is given by Profile 4. Arrow widths are proportional to AFT counts in  X  ‾ train,g(AFT). For N&undys graph, all the AFTs are represented (control situation). For the other graph, the AFTs that significantly changed compared to N&undys group (t-test and Benjamini Hochberg correction with $FDR < 0.05$) were filtered; we then ordered AFTs by compositional changes compared to N&Undys group (L2 difference on  W  ‾ train,g,i(AFT) H ij(AFT) X  ‾ train,g,j(AFT) computed on both groups) and kept the top 20 AFTs in order to highlight the main changes in microbiota composition.
