# Supplementary material for: Four functional profiles for fibre and mucin metabolism in the human gut microbiome
Source: Microbiome. 2023 Oct 20;11:231. doi: 10.1186/s40168-023-01667-y (PMC10588041; doi:10.1186/s40168-023-01667-y)
Supplement: Supplementary file 11 — Additional file 10. Metabolic exploration. A dynamical metabolic exploration is made available in html pages. Archives must be unzipped and the html file must be open in a web browser. • Profile AFT frequencies. HTML page sources Within-profile-dynamical-map.zip. • Metabolic shifts during CD and dysbiosis. HTML page sources Metabolic-shifts-CD-dys-map.zip. [file 40168_2023_1667_MOESM10_ESM.zip › Within-profile-dynamical-map.zip!/Profiles_characterization/slider.html]

 


Profile 1

Profile 2


Profile 3

Profile 4

Profile 1

**Within-profile KO and GH frequencies mapped on the metabolic network.** For each profile, i.e. each row of the profile matrix  
H(AFT) , we display the within-profile AFT frequencies, expressed for profile i and AFT j as  Hij(AFT)∑k ∈ KOHik(AFT) or Hij(AFT)∑k ∈ GHHik(AFT) depending if j is a KO or a GH. The resulting value is plotted in green and blue color scales for GH and KO respectively, with edge width proportional to the value. These plots represent the intrinsic functional potential of the different profiles, like Fig. 3.a and unlike Fig. S3 or 3.b which represent the profile contributions to the metagenome.

Profile 2

**Within-profile KO and GH frequencies mapped on the metabolic network.** For each profile, i.e. each row of the profile matrix  
H(AFT) , we display the within-profile AFT frequencies, expressed for profile i and AFT j as  Hij(AFT)∑k ∈ KOHik(AFT) or Hij(AFT)∑k ∈ GHHik(AFT) depending if j is a KO or a GH. The resulting value is plotted in green and blue color scales for GH and KO respectively, with edge width proportional to the value. These plots represent the intrinsic functional potential of the different profiles, like Fig. 3.a and unlike Fig. S3 or 3.b which represent the profile contributions to the metagenome.

Profile 3

**Within-profile KO and GH frequencies mapped on the metabolic network.** For each profile, i.e. each row of the profile matrix  
H(AFT) , we display the within-profile AFT frequencies, expressed for profile i and AFT j as  Hij(AFT)∑k ∈ KOHik(AFT) or Hij(AFT)∑k ∈ GHHik(AFT) depending if j is a KO or a GH. The resulting value is plotted in green and blue color scales for GH and KO respectively, with edge width proportional to the value. These plots represent the intrinsic functional potential of the different profiles, like Fig. 3.a and unlike Fig. S3 or 3.b which represent the profile contributions to the metagenome.

Profile 4

**Within-profile KO and GH frequencies mapped on the metabolic network.** For each profile, i.e. each row of the profile matrix  
H(AFT) , we display the within-profile AFT frequencies, expressed for profile i and AFT j as  Hij(AFT)∑k ∈ KOHik(AFT) or Hij(AFT)∑k ∈ GHHik(AFT) depending if j is a KO or a GH. The resulting value is plotted in green and blue color scales for GH and KO respectively, with edge width proportional to the value. These plots represent the intrinsic functional potential of the different profiles, like Fig. 3.a and unlike Fig. S3 or 3.b which represent the profile contributions to the metagenome.
